# Utilization of Glycosaminoglycans/Proteoglycans as Carriers for Targeted Therapy Delivery

**DOI:** 10.1155/2015/537560

**Published:** 2015-09-10

**Authors:** Suniti Misra, Vincent C. Hascall, Ilia Atanelishvili, Ricardo Moreno Rodriguez, Roger R. Markwald, Shibnath Ghatak

**Affiliations:** ^1^Department of Regenerative Medicine and Cell Biology, Medical University of South Carolina, Charleston, SC 29425, USA; ^2^Department of Biomedical Engineering/ND20, Cleveland Clinic, Cleveland, OH, USA; ^3^Division of Rheumatology & Immunology, Department of Medicine, Medical University of South Carolina, 114 Doughty Street, Charleston, SC 29425, USA

## Abstract

The outcome of patients with cancer has improved significantly in the past decade with the incorporation of drugs targeting cell surface adhesive receptors, receptor tyrosine kinases, and modulation of several molecules of extracellular matrices (ECMs), the complex composite of collagens, glycoproteins, proteoglycans, and glycosaminoglycans that dictates tissue architecture. Cancer tissue invasive processes progress by various oncogenic strategies, including interfering with ECM molecules and their interactions with invasive cells. In this review, we describe how the ECM components, proteoglycans and glycosaminoglycans, influence tumor cell signaling. In particular this review describes how the glycosaminoglycan hyaluronan (HA) and its major receptor CD44 impact invasive behavior of tumor cells, and provides useful insight when designing new therapeutic strategies in the treatment of cancer.

## 1. Introduction

During development, wound healing, and malignancies, normal cells and cancer cells often have to traffic through extracellular matrices (ECMs). ECMs form the microenvironment around cells and are composed of a dynamic and complex assortment of collagens, glycoproteins, glycosaminoglycans, and proteoglycans. Many cells can only migrate and grow in cultures when they are attached to surfaces through ECM. Some studies have reported that cancer cells can only traffic through the ECM via the proteolytic cleavage of structural barriers in ECM, while other studies have also indicated that neoplastic cells can traverse the ECM without mobilizing proteases [1, 2]. Thus, the ECMs produced by epithelial cells and stromal cells provide much more than just mechanical and structural support and are involved in the regulation of cell morphology, metabolism, differentiation, and survival.

**Figure 1:**
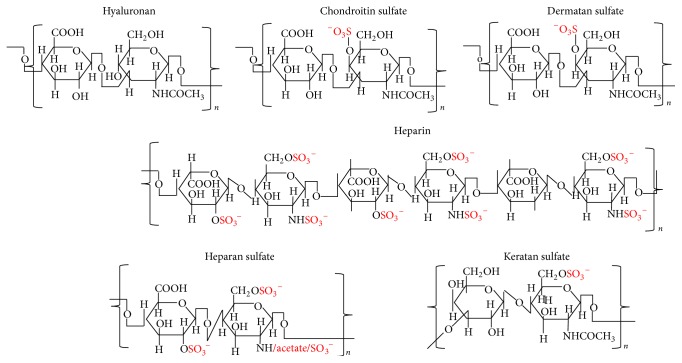
Structures of repeating disaccharides of glycosaminoglycans.

**Figure 2:**
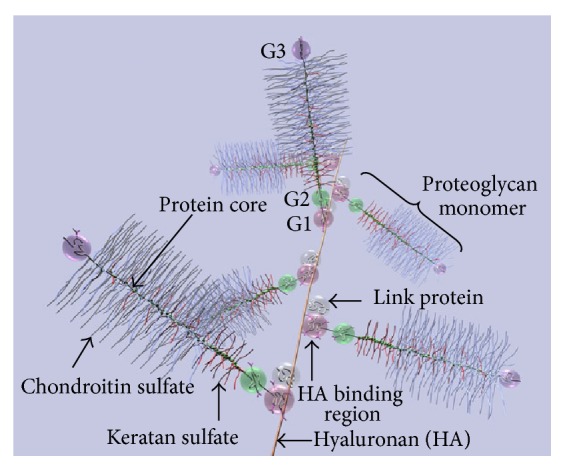
Diagram of part of an aggrecan aggregate. G1, G2, and G3 are globular, folded regions of the central core protein. Proteoglycan aggrecan showing the noncovalent binding of proteoglycan to HA with the link proteins.

**Figure 3:**
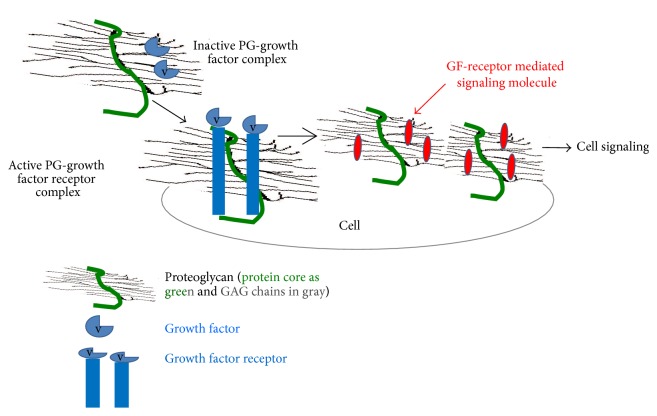
Proteoglycans act as coreceptors for growth factor receptor (GFR) signaling, thus influencing cell signaling and cell behavior. GAGs present as a part of proteoglycans on the cell surface and in ECM, bind to numerous proteins, and modulate their function.

Proteoglycans (PGs) (Figures 1 and 2) are proteins with a variable number of glycosaminoglycan (GAG) side chains [3]. The three classes of PGs with GAG chains and core proteins are (i) chondroitin/dermatan sulfate (CS/DS) PGs; (ii) heparin/heparan sulfate (Hep/HS) PGs; and (iii) keratan sulfate (KS) PGs [4, 5]. Hyaluronan (HA), a GAG, is synthesized without a core protein [6]. As indicated by their names (Figures 1 and 2), the GAGs other than HA are sulfated. GAGs have a critical role in assembling protein-protein complexes such as growth factor-receptor or enzyme-inhibitor interactions on the cell surface and in the extracellular matrix. These interactions can transduce signals by formation of ternary complexes of ligand, receptor, and PG for initiating cell signaling events or inhibiting biochemical pathways (Figure 3). Thus, GAGs can potentially sequester proteins and enzymes and present them to the appropriate site for activation. For a given high-affinity GAG-protein interaction, the positioning of the protein binding oligosaccharide motifs along the GAG chain determines if an active signaling complex is assembled at the cell surface or an inactive complex is sequestered in the matrix [7–9]. Overexpression of HA synthase 2 (HAS2) increases receptor tyrosine kinase-dependent signaling in breast and colon cancer cells [10–13], whereas antisense-mediated suppression of HAS2 inhibits tumorigenesis and progression of breast and prostate cancers [14, 15]. PGs and GAGs can have various physiological functions in different organs as well as roles in various pathologies. The details of these properties of GAGs and PGs are beyond the scope of this chapter. The present chapter will review the works that describe the utilization of GAGs in delivery of molecules for therapeutic purposes and highlights new possibilities for modulating HA interactions with CD44 variants (CD44v) for therapeutic control of cancer.

## 2. Biology of Hyaluronan and Its Receptor CD44

### 2.1. Biology of HA

HA is a major component in the ECM of most mammalian tissues, and HA accumulates in sites of cell division and rapid matrix remodeling that occurs during embryonic morphogenesis, inflammation, and tumorigenesis [16–19]. HA is found in pericellular matrices attached to HA-synthesizing enzymes or its receptors and is also present in intracellular degradation compartments [18–25]. HA is omnipresent in the human body and in all vertebrates, occurring in almost all biological fluids and tissues, with the highest amounts in the vitreous of the eye, synovial fluids, and the ECM of soft connective tissues. HA has repeat disaccharides consisting of D-glucuronic acid and* N*-acetyl glucosamine (Figure 1) [26–28]. Native HA has a very high molar mass, usually in the order of millions of Daltons (10^5^ to 10^7^ Da) before being progressively degraded into smaller fragments during its catabolism and eventual lysosomal degradation [27, 29, 30]. It possesses interesting viscoelastic properties based on its polymeric and polyelectrolyte characteristics. The amount present in tissues depends on its synthesis by synthases [31], degradation by hyaluronidases [32], and clearance through lymphatics (by LYVE-1 receptors) [33] and liver (by HARE receptors) [34]. Multivalent interaction of HA with CD44 collaborates in driving numerous tumor-promoting signaling pathways and transporter activities [35]. HA regulates proliferation and motility through CD44 and RHAMM (Receptor for HA Mediated Motility) [36], whereas the binding of HA to Intercellular Adhesion Molecule 1 (ICAM-1) may contribute to the control of ICAM-1-mediated inflammatory activation [37]. Despite its simple structure, HA is an extraordinarily versatile GAG and is involved in several key processes, including early EMT in development and morphogenesis, cell signaling, wound repair and regeneration, matrix organization, and many inflammatory pathologies [10, 13, 18, 19, 21–25, 38–47]. During carcinogenesis, changes in both nonnative CD44v (splice variants) and native (standard) CD44s arise [48]. Downstream signals of HA-CD44v interactions have been found to promote tumorigenesis, cancer cell migration, and metastasis [46, 47, 49]. Thus, various strategies are evolving for treatments of cancer that focus on HA and CD44, including interference with the HA-CD44v signaling, by either targeting drugs to CD44v [50], targeting drugs to the HA matrix [51], or interfering with HA-CD44v interactions [52].

### 2.2. HA Metabolism

Increased HA synthase (HAS) activity can contribute to tumor growth. 4-Methylumbelliferone (4-MU) has been widely investigated as a HAS inhibitor and has been shown to inhibit growth and motility and to induce apoptosis of several cancer cell lines.* In vivo*, 4-MU reduces tumor microvessel density [53], suppresses development of distant metastasis [54], and sensitizes human pancreatic cancer cells to the cytotoxic drug gemcitabine [55]. Although 4-MU may serve as an interesting anticancer approach in various cancers [53–55], no clinical trials have been conducted to use it as an anticancer activity because it is not patentable.

HA-degrading enzymes, hyaluronidases (HYALs), show both cancer-promoting and suppressing properties. In several clinical trials, the addition of bovine HYAL in therapeutic protocols resulted in slowing the growth of tumors [56, 57]. However, further studies have been abandoned due to induction of inflammation or pain in the joints because of enhanced HYAL activity in normal tissues [58].

The major concentrations of HA are found within the skin and musculoskeletal system, which account for 50% of total body HA. In these tissues, the rate of HA turnover is rapid (*t*1/2~0.5 d) by comparison with other extracellular matrix components [29]. Surprisingly, most degradation does not occur within the skin itself; rather HA is transported through the lymphatic system to distant lymph nodes where 90% of the HA in lymph is degraded or reenters the circulation to be rapidly endocytosed by liver endothelial HA receptors [59, 60]. Hence, lymph nodes are a major outlet for HA transport from tissues such as the skin and intestine, where they extract and metabolize as much as 10% of the total body HA content within a 24 h period [61–63] through the afferent lymph pathways where its larger polymers are more rapidly removed. This turnover can be further increased after either tissue injury or sepsis. The HA from lymph nodes enters the circulation resulting in a half-life of less than 1 minute (in rats) [64] and is distributed into the liver [65], where liver endothelium is a site of uptake and degradation of HA. The role and fate of HA have been widely investigated; however, effects of size and dose of HA on its metabolism have not been well documented yet. Similarly, enhanced accumulation of the matrix HA in the cortical renal interstitium is linked to the proinflammatory events in the kidney. It has been shown that HA-synthase and hyaluronidase genes are constitutively expressed in renal cells suggesting that HA synthesis as well as HA degradation occurs in these cells [66, 67]. In interstitial nephritis sites where HA is increased, HA receptor CD44 is expressed in high levels suggesting* in vivo* interaction of HA and CD44 [68, 69].

### 2.3. Biology of CD44

**Figure 4:**
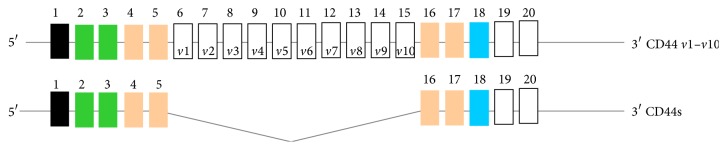
Alternative splicing in CD44 pre-mRNA. CD44 pre-mRNA is encoded by 20 exons. The common CD44s (hematopoietic) form contains no extra exons, and the protein may have a serine motif encoded in exon 5 that initiates synthesis of a chondroitin sulfate or dermatan sulfate chain. Alternative splicing of CD44 predominantly involves variable insertion of 10 extra exons with combinations of exons 6–15 and spliced in* v*1–*v*10 into the stem region, of which* v*3 encodes a substitution site for a heparan sulfate chain. Variable numbers of the* v* exons can be spliced in epithelial cells, endothelial cells, and inflammatory monocytes and also upregulated commonly on neoplastic transformation depending on the tissue.

To understand the role of the HA receptor CD44 in modifying malignant properties, it is essential to recognize its structures. Due to paucity of studies regarding involvement of CD44 variants in tumor angiogenesis and cancer stem cells, we discuss its functions in tumor cells in general and how cell-specific perturbation of HA-CD44v interaction can be used to inhibit cancer growth. CD44 (Figure 4) is a transmembrane protein encoded by a single gene. Due to alternative splicing, multiple forms of CD44v are generated that are further modified by N- and O-linked glycosylations. The smallest CD44 isoform that lacks variant exons, designated CD44s, contains an N-terminal signal sequence (exon 1), a link module that binds to HA (exons 2 and 3), a stem region (exons 4, 5, 16, and 17), a single-pass transmembrane domain (exon 18), and a cytoplasmic domain (exon 20). In all forms of CD44 cDNAs, exon 19 is spliced out so that the transmembrane domain encoded by exon 18 is followed by the cytoplasmic domain encoded by exon 20, producing the 73 amino acid cytoplasmic domain. CD44s can be a proteoglycan with a potential CS or DS substitution. Insertion of the* v*3 exon includes the potential for HS chain substitution [70], which can influence ligand binding and cell behavior by allowing CD44 to be a coreceptor for HGF with c-met [71]. The affinity of CD44 for these GAG substitutions depends on posttranslational modifications, such as modification in glycosylation [72] during alternate splicing of variant exons, and this function depends on cell type and growth condition. Isoforms that contain a variable number of exon insertions (*v*1–*v*10) at the proximal plasma membrane external region are expressed primarily on tumor cells [43, 70, 73].

Decades of research have shown that CD44 participates in major oncogenic signaling networks and in complexes with oncogenes that promote every aspect of tumor progression [18, 70]. Conversely, CD44 is extremely sensitive to changes in the microenvironment, and its reaction to changing extra- and intracellular conditions is still the subject of active research. For example, it was found that, in breast cancer cells, CD44 may act as a metastatic suppressor gene when it is influenced by reactive oxygen species (ROS), as seen by decreased CD44 protein expression in the Alpha5 cell line in a compensatory response to increased MnSOD protein expression [74]. Many of the contradictory findings published to date may be due to experimental and technical differences among studies; however, a picture has emerged suggesting that CD44 may function differently at different stages of cancer progression [75, 76]. For example, mice with germline disruptions of CD44 display relatively mild phenotypes compared with mice in which tissue-specific CD44 function is disrupted at adult phases of development or in later phases. This suggests that the absence of CD44 in early development and a loss of CD44 function late in development are tolerated differently [70].

## 3. Targeted Drug Delivery

Drug delivery can be localized, oral, or systemic by various carriers. Drug delivery systems that combine the benefits of various approaches will be made to address the needs of specific applications. Oral drug delivery is the easiest and a convenient route for drug administration. However, anticancer drugs, docetaxel and doxorubicin, and insulin in diabetes cannot be delivered orally because of their low oral bioavailability for various reasons. Nanoscale drug-delivery systems improve drug release kinetics, regulate biodistribution, and minimize toxic side effects, which enhances the therapeutic index. Nanoparticle diameters ranging from 70 nm to 200 nm demonstrate the most prolonged circulation times [77], minimizing the risk of recognition by the reticuloendothelial system as well as clearance by the liver and the spleen [78, 79].

GAGs are utilized in nanoscale drug delivery systems or in other formats, to deliver cargo, systemically or locally, loaded with drugs or biologics for therapeutic purposes to treat a variety of disease conditions, including cancer, glaucoma, wound healing, and burn. For wound healing and burn injury, the drugs are delivered by direct application of GAGs in conjunction with drugs. Ophthalmic drugs are also delivered locally. Systemically administered therapeutic molecules for cancer treatment enter into blood vessels of the tumors, pass the vessel wall, move through the ECM, avoid getting cleared by the lymphatics, and finally migrate through the interstitium barrier [80]. In reality, the case is not so simple. Use of intravital microscopy has revealed the complex anatomical barriers involved and has provided functional insights of their properties. These barriers can be abnormal, change with space and time, and depend on host-tumor interactions [81, 82]. The GAG carrier should have bioadhesive preference to tumor vasculature rather than to normal vasculature. Thus, the main focus of the delivery will be in the tumor vasculature. How will the drug penetrate the barrier that shields the tumor from the delivery? How can the surrounding blood vessels be targeted? How will the drug differentiate cancer cells from normal cells? How can the drug efflux following entry into cancer cells be avoided? Solutions of these and other questions are discussed below.

## 4. GAGs/PGs in Drug Delivery

### 4.1. Heparin/Heparan Sulfate in Drug Delivery

Heparin is primarily used as a blood anticoagulant due to its binding to the serine threonine protease antithrombin causing the inhibitor to inactivate thrombin [83]. Heparin was chosen as a carrier of Amphotericin B in heparin coated nanoparticles [84]. However, its use was discontinued because of its rapid clearance from blood due to heparin binding to hepatic liver sinusoids. Heparin still attracts attention due to its antiangiogenic and anticancer activities in addition to its activities on anticoagulation, deep vein thrombosis, and pulmonary embolism. Heparin is not absorbed orally because of its high molecular weight, negative charge, and hydrophilicity and therefore cannot be used in oral drug delivery [85]. By conjugating heparin with hydrophobic bile taurocholic acid (TCA), oral bioavailability of heparin was increased. For example, less water soluble docetaxel (DTX) was made orally available through conjugation with heparin and TCA, and as a result, excellent bioavailability of DTX increased [86]. In fact the conjugate is absorbed in the ileum by a bile acid transporter as well as by passive diffusion [87].

The high negative charge density of heparin has been exploited to form nanoparticles based on electrostatic interactions. Due to affinity for growth factors, heparin can potentially be a good carrier for these growth factors. Heparin nanoparticles were noncovalently assembled via electrostatic interactions between low-molecular-weight heparin (LMW-Hep) and* N*,*N*,*N*-trimethyl chitosan chloride (TMC). Vascular endothelial growth factor (VEGF) was entrapped in these LMW-Hep/TMC nanoparticles by affinity interactions with LMW-Hep. Controlled zero order release of VEGF from these nanoparticles was observed over a period of 14 days, and a total cumulative release was about 49% [88]. Heparin-Chitosan/c-PGA nanoparticles were prepared for multifunctional delivery of heparin and bFGF in ischemic tissues [89]. In addition to the physical incorporation of heparin into nanomaterials, heparin-decorated HA-based hydrogel particles were also prepared with consistent release of growth factors and bioactive HA molecules [90]. The HA was first modified with heparin and then formed into spherical nanoparticles that were used as vehicles for growth factor delivery in hydrogel matrices. For example, packing a covalently linked perlecan that contains HS chains with HA microgels was used as an injectable therapeutic agent to release BMP2 to repair cartilage matrix by intra-articular injection in a reversible animal model of osteoarthritis. The results show that a knee treated with the microgel had less damage than a control knee. The microgels act as a depot of BMP2, which is slowly released in the affected joint [91].

Depending on the type of HS-PG, the number of GAG chains varies from 1 to 100 attached to protein cores ranging from 10 to 500 kDa [92–94]. HS-PGs act as cell-surface endocytosis receptors [95]. The two major families of HS-PGs are the transmembrane syndecans and the GPI-anchored glypicans [7]. Recently, it was reported that syndecan-4, a ubiquitous transmembrane PG [96] present on the cell surface, binds and mediates the transport of a cationic cell penetrating peptide (CPP; 17 amino acids) into the cells [97]. CPP is a universal carrier that crosses the plasma membrane carrying drugs that include peptides, oligonucleotides, siRNAs, and liposomes [98–103]. The penetrating peptide can carry cargoes many times larger than its own size. Thus, application of CPPs appears to be encouraged in drug targeting and delivery, although the mechanism of translocation is largely unknown. Glypican 1 expression is upregulated in pancreatic cancer cells and surrounding fibroblasts, and the mitogenic response of pancreatic cancer cells to bFGF and HB-epidermal growth factor is abrogated by antisense attenuation of this HS-PG [104]. Similarly, perlecan expression is upregulated at sites of active angiogenesis, and the angiogenic effects of bFGF are suppressed by experimental downregulation of perlecan [105]. The HS-PGs found in the ECM (perlecan, aggrecan, and collagen XVIII) are large modular proteins that contribute to the structure, hydration, and permeability of the ECM. The capacity of HS-PGs to interact with both the matrix architecture and soluble ligands defines a unique combination of properties that enables normal cells to sense and respond to controlling influences in their environment. Cancer cells employ various mechanisms to exploit these properties to gain a survival advantage. Grubb et al. [106] used the high capacity uptake function mediated by HS-PGs to investigate if a lysosomal enzyme, beta-glucuronidase, could be taken up and transported to the lysosome as a possible way to treat lysosomal storage diseases. In these diseases, undigested GAGs collect progressively in lysosomes due to mutations that inactivate various enzymes required for complete degradation of GAGs, which leads to lysosomal dysfunction and manifestation of diseases associated with aging or other pathologies [107].

### 4.2. Chondroitin/Dermatan Sulfate in Drug Delivery

Use of biodegradable and nontoxic biomaterials from natural polymers has been explored to prepare materials for drug encapsulation and controlled delivery into tumors. Chondroitin sulfates have good biocompatibility and are primarily located on PGs in ECMs in tissues such as cartilage, skin, corneas, and umbilical cords. While circulating in the body during drug delivery, the hydrodynamic and hydrophilic properties of CS prevent undesirable interactions with plasma proteins and cells. Anaerobic bacteria,* Bacteroides thetaiotaomicron *and* Bacteroides ovatus*, which are residents of the large intestine, degrade CS. This suggests that CS can be a good candidate as a drug delivery agent to intestine for treatment of cancer. The positive charge on polyethylene imine (PEI) in DNA-PEI complexes can be neutralized by coating them with the highly anionic CS. A complex consisting of 10 kDa CS, PEI, and an expression vector for granulocyte macrophage-colony stimulating factor (GM-CSF) was able to deliver the plasmid into intraperitoneal and subcutaneous tumors in a syngeneic mouse model, and the released CSF inhibited tumor growth [108]. Water solubility of CS has limited its application as a drug-functionalized solid material. Hydrophobic modification of CS by O-acetylation was then used in a nanosize delivery model that increased bioavailability for anticancer drugs together with minimum side effects [109–111]. Nanosize also increased the potential for localization in tumors thus providing better targeting through enhanced permeation and retention (EPR) [112] in tumor vasculatures. CS nanocapsules, CS nanogels, and CS functionalized mesostructured silica nanoparticles have been developed for controlled release and targeted delivery of bioactive compounds. Nanocapsules have relatively lower density, less consumption, high loading capacity, and better protection of the loaded compound. Nanogels are comprised of hydrophobic inner shell cores and hydrophilic outer shells or coronas. The hydrophobic cores determine drug loading efficiency and release. The hydrophilic coronas provide prolonged and more effective circulation of the relevant drug and can act as barriers against interactions with other cells, proteins, and body tissues [113, 114], which prevents recognition by the reticuloendothelial system.

Melanoma chondroitin sulfate proteoglycan (MCS-PG) is another example that can be a target to induce apoptotic signals in melanoma cells. MCS-PGs are expressed in 85% of melanomas [115], and they increase metastatic properties motility [116] and invasion [117] in melanoma cells and promote growth of the cells. However, they are also present in cells of the melanocyte lineage, in basal cells of the epidermis, in hair follicles, and in pericytes [115]. Therefore, the melanocytes would also be exposed to both anti-MCS-PG monoclonal antibodies and immune-toxin mediated attack strategies [118, 119]. Xi et al. [120, 121] prepared a CS nanocapsule loaded with indomethacin for delivery and demonstrated its pH responsiveness when studied* in vitro* in a pharmacological drug release model. Xi et al. [121] also prepared functionalized mesoporous silica nanoparticles (MSNs) with CS coronas (NMCS-MSN), which exhibited high drug loading capacity and pH-triggered controlled release of drug simultaneously. Typically, NMCS-MSN was mixed with test drug doxorubicin to form NMCS-MSN-Dox that was then cross-linked with bisulfate. This resulted in release of doxorubicin in a pH responsive manner that was more cytotoxic to HeLa cells than free doxorubicin. Nanogels are self-organized when a solution of acetylated CS (AC-CS) in dimethylsulfoxide (DMSO) is dialyzed in aqueous medium. DMSO is displaced by water, and nanogels are formed. Park et al. [122] prepared doxorubicin loaded AC-CS nanogels by dialyzing a mixture of doxorubicin and AC-CS in DMSO in borate buffer. Another notable application of CS in a drug delivery vehicle is the use of folate linked CS on surfaces of Pluronic 127 nanogels [123] to target folate receptor rich cancer cells. The Pluronic 127 is an inhibitor of the drug efflux transporter. This vehicle can release the drug payload following receptor mediated entry into folate receptor rich tumors, which then blocks the efflux of the drug and retains it inside the cells.

A form of DS known as DS 435 [124] with an average molecular weight of 22.2 kDa was prepared from beef lung mucosa. It has an unusual property to target neovascular transport. It contains 2-sulfated IdoUA and 4-sulfated GalNAc (with some 4,6-di-sulfated GalNAc) disaccharide structures, which do not bind to the antithrombin III receptor present on endothelium and which have low anti-Xa anticoagulant and fibrinolytic activities. These sulfation patterns on DS 435 selectively target the neovascular system and penetrate into the tumor matrix. Its subsequent uptake by tumors is high owing to its high heparin cofactor II binding. Nanoparticles with doxorubicin cores coated with DS 435 were prepared by high pressure homogenization. The particles injected intravenously showed deeper matrix penetration than regular doxorubicin. DS-Dox demonstrated better therapeutic efficacies in a human MX-1 breast xenograft study [124]. It was concluded that DS drug combinations were transported rapidly and selectively across tumor neovascular endothelium and penetrated deeply into the tumor matrix.

### 4.3. Keratan Sulfate (KS) in Drug Delivery

Keratan sulfate (KS) is a linear GAG that consists of repeating disaccharides composed of galactose and N-acetylglucosamine (GlcNAc) with sulfation at the 6-O positions on the disaccharides. KS, the major GAG in cornea, is present on one of three core proteins (lumican, keratocan, or mimecan) forming KS-PGs [125]. KS has long been believed to play a central role in corneal homeostasis because it is altered when the cornea becomes opaque during injury or disease [126]. Large quantities of cell-associated KS are found in the endometrial lining during the menstrual cycle and have an important role during embryo implantation. Specifically, the antiadhesive properties of KS have important roles in both endothelial cell migration in cornea epithelium and in embryo implantation during the menstrual cycle [127–129]. Several reviews describe possible structures and functions of KS-PGs [130–132], but the role of KS-PGs in drug delivery is not known clearly. KS plays an important role in mucopolysaccharidosis IVA (MPS IVA, Morquio A disease), an inherited lysosomal storage disorder that features skeletal chondrodysplasia. In this Morquio A syndrome, serum KS increases due to deficiency of N-acetylgalactosamine-6-sulfate sulfatase (GALNS), which is required to degrade KS. Recent studies used a bone-targeting drug delivery system with E6-tagged GALNS where GALNS was bioengineered with the N-terminus extended by the hexaglutamate sequence (E6) to improve targeting to bone (E6-GALNS). The results showed improved efficacy of enzyme-replacement therapy to bone and cartilage in Morquio A disease [133].

### 4.4. HA in Drug Delivery

#### 4.4.1. HA in Transdermal Delivery

Skin is the largest organ in the body (15% body weight), which contains most of the body's HA, and is a barrier against water loss and invading pathogens. The outer layer stratum corneum (15 *μ*m) provides this barrier function. Below the stratum corneum is epidermis (130–180 *μ*m), and next to it is the dermis (2000 *μ*m) that contains nerves, blood vessels, nociceptors, lymph vessels, hair follicles, and sweat glands. In normal skin, HA is found in the intercellular spaces of epidermis except in the upper granular layer and in the stratum corneum. Because HA is nonantigenic, intradermal delivery of substances using HA as a vehicle will not pose any problem. HA is used as a transdermal drug delivery vehicle in various forms, including hydrogels, and as microneedles to deliver insulin and immunogens for immunization. For example, the transdermal delivery of insulin using microneedles (MNs) fabricated with 15% HA containing insulin is used in diabetes patients [134]. In diabetic rats, the microneedles applied in skin dissolved in an hour with rapid release of insulin, and the holes produced by the microneedles disappeared within 24 hours demonstrating reversible skin damage. The insulin administered by this approach was almost completely absorbed from the skin into the systemic circulation as indicated by pharmacodynamic and pharmacokinetic parameters [134]. Similarly, during vaccination, transcutaneous immunization via these micro needles can be used for easy delivery of the antigens [135–138]. Recent advancements in self-dissolving microneedles using biodegradable materials is a promising vaccination approach [139–142]. For example, in transcutaneous immunization (TCI) of tetanus toxoid (TT) and diphtheria toxoid (DT), sodium HA was mixed separately with TT and DT to form the micro needles [139–142]. TCI systems with microneedles physically penetrate the stratum corneum and directly deliver the antigen into the epidermal layer and dermis, which contain an advanced immune system with antigen-presenting cells (APCs) such as Langerhans cells (LCs) or dermal dendritic cells (dDCs). The antibody titers against the toxoids increased, and the vaccination protected the TT-vaccinated animals against tetanus toxin.

Despite the high molecular weight and hydrophilicity of HA, it is known to be delivered through the skin tissue in both mice and humans using MNs [143–145], whereas in delivery of antibodies, the HA is composed of low molecular mass of 55 kDa and of 25 kDa [146]. MNs fabricated from HA show complete dissolution within 1 h of application to rat skin* in vivo* [134], whereas the delivery time of TCI (influenza vaccination)/TT/DT fabricated with HA is 1–5 min [141].

#### 4.4.2. HA in Burn Injury

Burn injury to skin and flesh is caused by heat or electricity. Depending on the depth of burn, injuries are classified in terms of degrees, from superficial (first-degree), to underlying layers (second-degree), and to all layers (third-degree). Burn injuries over relatively small areas are an example of an acute, local inflammatory state. Following such an initial burn injury, the affected skin/flesh progresses to tissue ischemia and necrosis [147]. The initial injury induces a zone where protein denaturation, significant tissue loss, and cell death occur. Burned tissue is in a cytotoxic and degenerative state [148]. Thus, proinflammatory mediator levels of tumor necrosis factor alpha (TNF-*α*), interleukin-1*β* (IL-1*β*), and interleukin-6 (IL-6) increase both systemically and locally in burn areas [149]. These mediators establish the pathophysiological environment of burns. Therefore, neutralizing these targets could effectively regulate the complex inflammatory cascade. Antibodies against TNF-*α* or IL-1*β* conjugated to high molecular weight HA diffuse slowly thus providing a sustained delivery of the antibodies in the wound [150]. Animal studies indicated that, in a partial-thickness rat burn model, HA treatment alone reduced burn progression by nearly 30%, while anti-TNF-*α*-HA reduced it approximately 70% and decreased macrophage infiltration into the injury site compared to controls [151].

#### 4.4.3. HA in Ocular Drug Delivery

HA was first discovered in the vitreous body by Meyer and Palmer in 1934 [26]. HA is a component of vitreous humor, lacrimal gland, conjunctiva, corneal epithelium, and tear film [152]. Graue et al. [153] were the first to create a novel noninflammatory preparation of HMW HA on a commercial scale, and its use in ocular surgery protects the corneal endothelium. The properties of HA that have made it a first choice in ocular drug delivery are excellent moisturization properties, mucoadhesiveness, extended drug-retention time in tear fluid and drug contact time [154], bioavailability and tissue healing properties, promotion of growth of corneal epithelial cells [155, 156], and suppression of inflammation [157, 158]. Numerous topically administered preparations have been developed based on these properties of HA, including pilocarpine [159–161], tropicamide [162], timolol [160, 163], and tobramycin [163]. Recently* in situ* gel technology has been developed in which modified HA solutions with drugs can be dropped into the conjunctival sac where it is converted into a gel with increased bioavailability and duration, which is a promising approach [164]. In this method, ophthalmic drug was delivered using graft copolymers prepared by coupling mono amine-terminated poloxamer (MATP) with a HA backbone using 1-ethyl-3-(3-dimethylaminopropyl)-carbodiimide (EDC) and* N*-hydroxysuccinimide (NHS) as coupling agents. This poloxamer-graft-HA hydrogel slows down drug elimination by lacrimal flow, both by undergoing* in situ* gel formation and by interacting with the mucus. Also, this hydrogel can be used to enhance wound healing of an injured mucus layer of the eye [164]. Noncancer drug delivery applications of GAG delivery systems are presented in Table 1.

### 4.5. Cancer Drug Delivery Applications of GAG/HA Delivery Systems

#### 4.5.1. Role of HA and CD44 in Drug Conjugates

Tumors often exhibit a phenomenon known as the enhanced permeability and retention effect (EPR). This is a result of two issues: increased permeability of the capillary endothelium in malignant tissues compared to that of normal tissues and the lack of tumor lymphatic drainage within the tumor interstitium that results in drug accumulation. If a therapeutic agent is coupled to a suitable biodegradable drug carrier, then such carriers have the potential of increasing the concentration of drug distribution in the tumor tissue due to the EPR effect [44]. Administering a drug in a carrier alters its distribution profile by directing it into the tumor and by exploiting the EPR effect [18]. The therapeutic efficacy depends on a specific drug target process that has the ability to cross the cellular barrier, find the tumor, and reach the intracellular target sites to alter the tumor biology. Thus, an appropriate drug carrier would effectively transport the drugs through the cellular membrane with improved drug circulation time, solubility and stability of the drug, and would reach the cell and intracellular target sites. Several interesting smart carrier systems based on both synthetic and natural polymers have been designed and developed to achieve these goals [165].

In recent years, several features in HA prompted investigators to consider it as an important driver for drug delivery for the following reasons. (a) HA is a promising component from the pharmaceutical standpoint because it is biodegradable, biocompatible, nontoxic, hydrophilic, and nonantigenic. (b) The carboxylate on the glucuronic acid and the hydroxyls on the* N*-acetyl glucosamine can be potentially used to conjugate a drug [166], and the acetyl on the* N*-acetyl glucosamine can be removed enzymatically to expose an amino group that can also be used for drug conjugation [167]. (c) The molecular mass of HA is an important consideration while preparing the drug conjugate. For instance, a 90 kDa fluorescein-labeled HA gradually accumulated in the liver and then was distributed into endothelium, whereas 10 kDa or less HA was rapidly excreted into urine* in vivo* [168]. This indicates that HA with high average molecular weight has hepatic targeting ability. The success of HA as a carrier depends on the number of receptors available on the target cells and on the affinity between the homing ligand and the receptor. HMW HA can cross-link with a number of CD44 receptors and be endocytosed. However, it is rapidly cleared from circulation by the liver hepatocytes [169], and any excess of the targeting compound can lead to adverse effects [170]. This rapid clearance was circumvented by choosing HA oligosaccharides long enough to bind to CD44 but too short to bind to the HARE receptor, which may permit targeting to cells that overexpress CD44. The minimum HA length required to interact with individual CD44 molecules is 6 to 10 saccharides [171] with moderate affinity. Several preclinical studies have shown that HA chemically conjugated to cytotoxic agents improved anticancer properties of the agent* in vitro* [50, 172, 173]. HA-drug conjugates also improve the inadequate water solubility of some anticancer agents [18, 165, 174, 175]. Targeting CD44 presents a very promising and novel approach against HA-induced tumorigenesis.

#### 4.5.2. Approaches for Targeting HA

*(i) HA Backbone Based Conjugated Drugs*. HA conjugated drugs are more soluble in water than the drugs alone. For instance, the antimitotic chemotherapeutic agent paclitaxel (PTX) has low water solubility. Upon conjugation to HA, water solubility of the prodrug HA-PTX significantly increased, and CD44 dependent cellular uptake increased* in vitro* [176]. The prodrug HYTAD1-p20 (HA-PTX) conjugate (renamed as ONCOFID-P by the pharmaceutical company Fidia) shows significant benefit over original PTX in terms of* in vitro* activity against bladder carcinoma cells,* in vivo* safety profile, and pharmacokinetics [177]. ONCOFID-P is in Phase II clinical study in the intravesicle therapy of patients with nonmuscle invasive cancer of the bladder (EudraCT 2009-012274-13). A phase I clinical study was initiated to investigate its maximum tolerated dose and safety profile following i.p. infusion in patients affected by intraperitoneal carcinogenesis in ovarian, breast, stomach, bladder, or colon cancers [178–180]. Luo et al. coupled PTX-N-hydroxysuccinimide ester (PTX-NHS) with HA of molecular weight ~11 kDa [181]. PTX release from the hydrogel film was evaluated* in vitro* using selected antibacterial and anti-inflammatory drugs [181]. The pharmaceutical company Fidia prepared ONCOFID-S, another HA prodrug conjugate with SN-38, the active CPT11 (irinotecan) metabolite. The HA used had a molecular weight of ~200 kDa.* In vitro* and* in vivo* cytotoxicities were investigated using ONCOFID-S in several CD44 overexpressing cancer cells, including colon, gastric, breast, esophageal, ovarian, and human lung cancer cells [178]. The treatments reduced all parameters correlating with poor prognosis in peritoneal colorectal cancer (CRC) carcinomatosis without any myelotoxicity or mesothelial inflammation [182]. A recent* in vivo* study with HA-cisplatin reported a significant improvement in antitumor efficacy, with lower toxicity compared to standard cisplatin, in locally advanced head and neck squamous cell carcinoma [183].

*(ii) HA-Encapsulated Drugs*. Another strategy for HA-based CD44 targeting utilizes the concept that the large volume domain of HA (molecular weight > 750 kDa) can noncovalently entrap small therapeutic molecules within this matrix. HA was then used as a macromolecular carrier for the irinotecan drug along with its targeting properties [184]. The principle is that the HA-drug complex will accumulate in the microvasculature of the xenograft tumor, increase drug retention at the tumor site, and allow for active uptake of the drug by the tumor cells via HA receptors. Clinical trials of three such HA formulations (termed hyaluronic acid chemotransport technology (HyACT)) have been undertaken in Australia. Phase I clinical evaluation of two formulations based on HA (HyACT) with 5-fluorouracil (5-FU) (HyFIVETM) and on HA with Dox (HyDOXTM) demonstrated reasonable efficacy without compromising safety of these formulations [185, 186].

*(iii) HA-Tailed Drug Carriers*. These include the following HA conjugates.

- Bisphosphonates (BPs) [187]: high molecular weight HA is linked via a hydrazide group to bisphosphonate (HA-BP). The hydrazide group of the HA-BP can also be used to explore hydrazone linkage of other drugs, such as doxorubicin, which could be integrated into the hydrogel matrix following treatment with aldehyde modified-HA [187]. The cytotoxicity of the HMW HA-BP was directly proportional to cell surface HA receptor levels.
- Carbonates [188, 189]: Di Meo et al. proposed a bioconjugate (HA-pCB) composed of n-propyl carbonate linked to HA via an ester linkage. Its cellular uptake was evaluated* in vitro* on a variety of human tumor cells. HA-pCB produced high intracellular accumulation of boron atoms.
- Chitosan [190, 191]: Jain et al. prepared chitosan-HA nanoparticles (HA-CTNPs). 5-FU loaded nanoparticles were then prepared by the ionotropic gelation method. Their surface was coupled to the carboxylic group of HA by using carbodiimide chemistry [1-ethyl-3-(3-dimethylaminopropyl) carbodiimide (EDC)] with chitosan amine groups to form amide linkages. The* in vitro* cellular uptake and cytotoxicity results suggest that the 5-FU loaded HA-CTNP formulation leads to increased uptake in comparison with chitosan nanoparticles alone (CNTPs), and that this significantly enhanced cytotoxicity compared with either CNTP or free 5-FU in HT29 colorectal cancer cell lines, which overexpress the CD44 receptor.
- Gagomers (GAG-mers) [192]: GAG-mers (glycosaminoglycan cluster of particles) are composed of lipid molecules that self-assemble into particulate clusters in hydrophilic solutions, which are then covalently coated with HMW (1.2–5 MDa) HA by carbodiimide activation of carboxyl groups at a lipid : HA ratio of 10 : 1 (w/w). When tested in primary head and neck cancers and normal cells taken from the same patient [192], GAG-mers selectively bound only to the tumor cells.
- Liposomes/lipoplexes [193–197]: HMW HA was decorated on nanosized encapsulated anticancer drugs. In a first study, mitomycin C (MMC) was encapsulated in HA-liposomes (HA-LIP), and their behaviors* in vitro* and* in vivo* were compared to those of plain liposomes. The cytotoxic activity of the drug loaded into HA-LIP was found to be ~100-fold that of free drug in cultured tumor cell lines overexpressing the HA receptors, but not in cells with low receptor expression levels.* In vivo* tumor model studies confirmed the higher antitumor activity of HA-LIP containing MMC compared with MMC alone or with unmodified liposomes. In a second study, complexes were prepared with plasmid DNA pCMV-luc and lipoplexes (conjugates), which contained HA and the amino-reactive group of a lipid, dioleoylphosphatidylethanolamine (HA-DOPE), to form cationic liposomes having negative zeta potential and a mean diameter of 250–300 nm [195]. With this approach, conjugation depends on the molecular weight of HA. The HA-LIP conjugate can be used to deliver plasmid DNA and siRNA to CD44 positive cancer cells [195, 198]. The presence of HMW HA in the lipoplexes enhanced nucleic acid protection from degradation by DNase I or RNAse VI. In case of LMW HA, the HA was linked to PE to form a conjugate in which only one PE molecule is linked to a HA molecule [196, 197]. This procedure enables a controlled amount of HA to be introduced into the liposomes. The ability to target LMW HA decorated liposomes was demonstrated in an interesting approach [196]. HA oligosaccharides were attached to PE and incorporated into the liposomes, which increased their recognition, cytotoxicity, and transfection efficiency by tumor cells expressing high levels of CD44 in a temperature-dependent manner. Uptake of the liposomes was also dependent on the density of HA.
- Micelles: the hydrophilic backbone of HA was first dissolved in DMSO and then conjugated via its carboxyl groups to amino functions of poly-L-histidine (PHis), or polyethylene glycol (PEG). These HA construct form nanocomplexes by self-organizing into micelles, and they can carry anticancer drugs, including paclitaxel (PTX). PTX when entrapped into the hydrophobic cores of the folic acid- (FA-) conjugated HA-C18 micelles exhibited higher cytotoxic activity compared to Taxol in MCF-7 cells that overexpress both the folate receptor and CD44. FA-HA-C18 micelles showed low cytotoxic activity compared to Taxol in A549 cells that only overexpress CD44 [199]. The HA-PTX [199], HA-DOX [200], and HA-salinomycin [201] micelles exhibited more pronounced cytotoxic effects on HA receptor overexpressing cancer cells than on receptor deficient cells.
- Nanocarrier: HA when conjugated to a nanocarrier acts as a protective structural component and provides a targeting coating that influences the circulation time and biodistribution (pharmacokinetic properties) and the cell specific uptake properties of the large carriers. Cargo liposomes or nanoparticles were able to deliver anticancer drugs, including epirubicin [50], doxorubicin [196], paclitaxel [176], and mitomycin-C [50], as well as siRNA, to CD44 overexpressing cells [202].
- In addition to the well-developed strategies described above, several multifunctional nanocompounds have recently been developed that combine therapeutic and diagnostic properties. These nanoparticles include quantum dots [203], carbon nanotubes [204], nanodots [205], graphene [206], gold nanoparticles [207], iron oxide nanoparticles [208], and silica nanoparticles [209], and they have been found to acquire novel characteristics after their conjugation with HA [203–210]. Cancer drug delivery applications of GAG/HA delivery systems are presented in Table 2.

#### 4.5.3. Approaches for Targeting CD44

CD44 proteins exist in three states with respect to HA binding: nonbinding, nonbinding unless activated by physiological stimuli, and constitutive binding [171, 211, 212]. After the HA-drug conjugates are internalized via CD44 [213], the drug can be released and activated mainly by intracellular enzymatic hydrolysis [176]. CD44 is endogenously expressed at low levels on various cell types in normal tissues [214], but it requires activation before it can bind to HA. Activated CD44 is overexpressed on solid tumors, but much less, or not at all on their nontumorigenic counterparts. Cellular activation can affect HA targeted drugs by inducing transition of CD44 to a high-affinity state. For example, tumor-derived cells express CD44 in a high-affinity state that is capable of binding and internalizing HA. Transitions from the inactive, low-affinity state to the active, high-affinity state by CD44 require posttranslational modifications. Posttranslational modifications of CD44 can be induced by ligation of antigen receptors [215], sulfation, or the action of cytokines [216]. Glycosylation is required for CD44 to bind HA on certain cell types, while glycosylations rich in sialic acid decrease HA binding [72, 217]. HA induces signaling when it binds to constitutively activated CD44 variants (CD44v) [218–221]. CD44 can also react with other molecules, including collagen, fibronectin, osteopontin, growth factors, and matrix metalloproteinases (MMPs), but the functional roles of such interactions are less well known [70]. Thus, targeting drugs to CD44 are one of the appropriate strategies for cancer treatment. HA is the major CD44 ligand, and HA with innate ability as a drug carrier increases the drug concentration on CD44 overexpressing cancer cells, as well as for other pathologies [166, 172, 181, 222–227]. HA-CD44 interaction, which deserves particular attention, can initiate signal transduction pathways leading to cancer cell growth, adhesion, migration, invasion, and metastasis. Therefore, inhibiting HA-CD44 interaction has been investigated [18, 19, 44, 228–230].

A considerable number of studies indicate that CD44 isoforms correlate with bad prognosis in patients with most human cancers [231–238] except in neuroblastomas and prostate cancer [239, 240]. CD44v6 is quite likely to be a suitable target for anticancer therapy because it is (a) causally involved in metastasis of a rat pancreatic carcinoma [241]; (b) redundantly correlated with the human tumors mentioned above; and (c) correlated with oncogenic functions in colorectal cancer (CRC) both* in vitro* and* in vivo* [12, 18, 43, 232, 234, 235]. Four approaches have been proposed to target CD44: (i) targeting with anti-CD44 antibodies, (ii) interrupting HA-CD44 interaction by HA oligosaccharides (oHAs), (iii) enzymatic degradation of HA, and (iv) tissue-specific deletion of CD44 variant signaling by our validated tissue specific delivery of CD44v6shRNA into tumor cells. Therapeutic applications of disrupting the HA/GAG biological systems are presented in Table 3.

*(i) Targeting with Anti-CD44 Antibodies*. Anti-CD44 antibodies against highly expressed CD44v variants can effectively target drugs to cells expressing a selective CD44v, which can then inhibit and disrupt CD44 matrix interactions and alter CD44 signaling and cause apoptosis [256]. Antibodies against highly expressed variants can also be designed to selectively deliver a cytotoxic drug to cancer cells. Anti-CD44v6 conjugated with a cytotoxic drug mertansine has been used in early phase clinical trials. In head neck and squamas cell cancer (HNSCC) patients, humanized anti-CD44v6 monoclonal antibody (HAMA) labelled with technetium-99 m was first tested [231]. Given the promising results of a phase I clinical study with the radionuclide-antibody conjugates [242–246], a new strategy was advanced to prepare an immunotoxin with bivatuzumab [247] and used in the next clinical trial on thirty HNSCC patients. Only three patients showed a partial response [257], and one patient died of toxic epidermal necrolysis, after which further trials were abruptly withdrawn. Later, HNSCC patients suffering from early stage breast cancer were also treated with this humanized CD44v6 antibody [258], and it accumulated in nontumor areas, indicating limitations in the use of this antibody therapy. Thus, CD44v6 remains a crucial target for tumor therapy. To address this issue, we have developed a novel tissue specific shRNA delivery strategy by a Cre-lox system. This technology is discussed below.

*(ii) Interrupting HA-CD44 Interaction*. This approach involves substituting multivalent interaction of HA with CD44 by monovalent interaction of HA-CD44 using small HA oligosaccharides (6–18 saccharide units (oHAs)) [248, 259]. The oHAs inhibit HA-CD44 downstream cell survival and proliferation pathways, and they stimulate apoptosis and expression of phosphatase and tensin homologue (PTEN) [41]. The oHAs also sensitize cultured cancer cells to some chemotherapeutic drugs by inhibiting expression of MDR1 and other ABC transporters [11, 13, 249]. While oHAs inhibit the growth of several tumors implanted as xenografts [41], they did not give consistent significant growth inhibition in adenoma growth of Apc Min/+ mice. Thus, we developed small interfering RNA (siRNA) and, even more advantageous, short hairpin RNA (shRNA), to target CD44v6 in colon cancer, showed that they can successfully interrupt HA-CD44v6 interaction and signaling (~90–95%). We then developed a novel shRNA delivery approach to target HA-CD44v6 specifically in tumor cells [18, 43, 44, 255], which is discussed in the following sections. Targeting CD44v6 with CD44v6shRNA inhibits distant tumor growth in mice, suggesting that it can also work against metastatic cancer cells. Due to its specificity for CD44v6, normal cells expressing CD44 remain unaffected [18, 43, 44, 255].

*(iii) Enzymatic Degradation of HA*. Hyaluronidases (HYALs) are a class of enzymes that predominantly degrade HA. However, HYALs can also degrade chondroitin sulfate and chondroitin [32]. They are endoglycosidases that hydrolyze the *β*-*N*-acetyl-D-glucosaminidic linkages in the HA polymer. Among the 6 HYALs present in the human genome HYAL-1, HYAL-2, and PH20 are well characterized. Recently, Lokeshwar et al. have shown that the expression of HYAL-1-*v*1 in bladder cancer cells that express wild type HYAL-1, induces G2-M arrest and apoptosis [252]. It has been shown that commercial bovine testicular or bacterial hyaluronidase acts as an antiadhesive compound on EMT-6 tumor spheroids [250], and hyaluronidase-disaggregated EMT-6 spheroids were shown to possess chemosensitivity to cyclophosphamide [250], and also to improve the therapeutic effectiveness of these agents, that is, by increasing the accessibility of solid tumors to the chemotherapeutic drugs. Unlike EMT-6 cells, Hyals have limitations as an antiadhesive agent for other human tumors [251].

*(iv) Tissue-Specific Deletion of CD44 Variant Signaling*. This section discusses the fundamental aspects of therapeutic cell-specific delivery addressing: (a) what to deliver (engineered therapeutic CD44v6shRNA delivery for* in situ*), (b) how to deliver (delivery strategies, in particular nonviral transferrin- (Tf-) coated PEG-PEI (Tf-PEG-PEI-nanoparticles) for* in situ* cell specific therapy), and (c) where to deliver (tumor-cell targets, in particular colon tumor cells for* in situ* cell-specific therapy). The technique of using shRNA in an expression vector is an alternative strategy to stably suppress selected gene expression, which suggests that the use of shRNA expression vectors holds potential promise for therapeutic approaches for silencing disease causing genes [260]. There are two ways to deliver shRNA in cancer cells, either using a viral vector or a nonviral vector. Viral vectors have been used to achieve proof of principle in animal models and, in selected cases, in human clinical trials [261]. Systemic targeting by viral vectors towards the desired tissue is difficult because the host immune responses activate viral clearance. Systemic administration of a large amount of adenovirus (e.g., into the liver) can also be a serious health hazard and even caused the death of one patient [261]. Nevertheless, there has been considerable interest in developing nonviral vectors for gene therapy. In this regard, nonviral vectors, such as positively charged polyethyleneimine- (PEI-) complexes shielded with polyethylene glycol (PEG), can be used safely to avoid the nonspecific interactions with nontarget cells and blood components [253].

**Figure 5:**
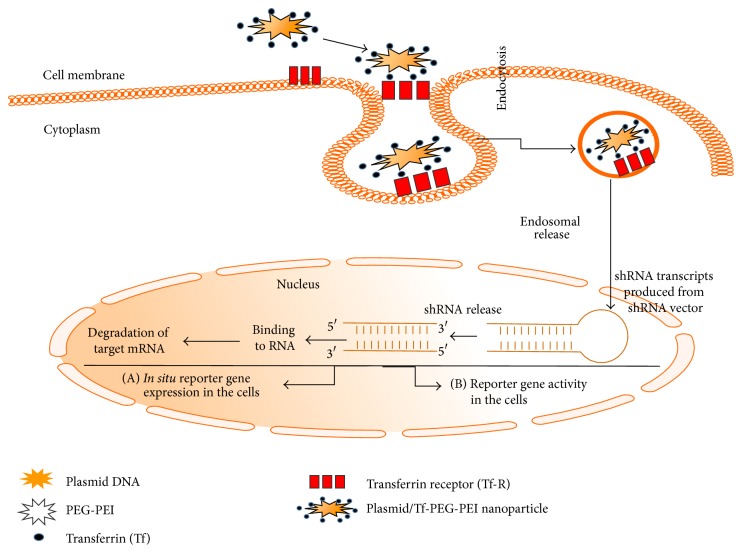
Schematic illustration of cellular uptake of plasmid DNA/Tf-PEG-PEI (nanoparticles) polyplexes, their shielding from nonspecific interaction, and the mechanism of action of shRNA (adapted from [43]). Internalization of PEG-shielded and Tf-R-targeted polyplexes into target cells occurs by receptor-mediated endocytosis after association of polyplex ligand Tf to Tf-R present on the target cell plasma membrane. Internalized particles are trafficked to endosomes followed by endosomal release of the particles and/or the nucleic acid into cytoplasm. Released siRNA will form a RNA-induced silencing complex and will be guided for cleavage of complementary target mRNA in the cytoplasm. siRNA (antisense) guide strand will direct the targeted RNAs to be cleaved by RNA endonuclease. Finally plasmid/Tf-PEG-PEI-nanoparticles delivery in the target cell shows reporter gene expression and activity.

**Figure 6:**
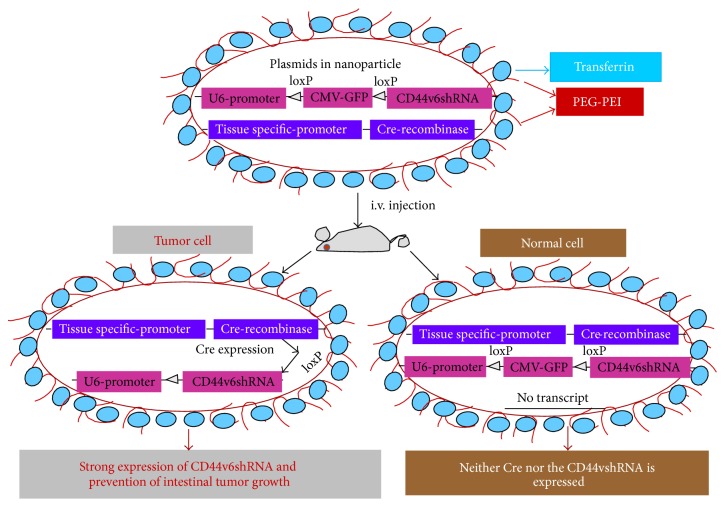
Model for delivery of shRNA (adapted from [18]). This illustration depicts cellular uptake of plasmid Tf-PEG-PEI nanoparticles and the mechanism of action of shRNA. First, a pSico vector containing a U6 promoter-loxP-CMV-GFP-STOP signal-loxP-CD44vshRNA (gene of interest) is made. Second, an expression vector with the Cre-recombinase gene controlled by the tissue specific promoter is created. Third, the two vectors are packaged in transferrin (Tf) coated-PEG-PEI nanoparticles that bind with Tf-receptors (Tf-R) present at high levels in the targeted tumor cells. Delivery of the vectors in normal and malignant cells from the targeted tissue results in deletion of the Stop signal and transcription of Cre-recombinase driven by the tissue specific promoter. The target gene (CD44vshRNA) is then unlocked and transcribed through the strong U6 promoter for high expression. The normal tissue cells are not affected because they do not make the targeted CD44 variant. Tf-PEG-PEI nanoparticle coated plasmids (pSico-CD44v6shRNA/pFabpl-Cre) circulating in blood accumulate at tumor regions enhanced by the EPR effect. Endocytosis mediated by ligand-receptor interactions occurs because the nanoparticles are coated with the Tf-ligand for the Tf-R receptor on the tumor cell surface.

Figures 5 and 6 illustrate the model for the uptake of Tf-PEG-PEI-nanoparticles carrying multiple functional domains. Nonviral vectors were once limited for their low gene transfer efficiency. However, the incorporation of various ligands, such as peptides, growth factors, and proteins, or antibodies for targets highly expressed on cancer cells, circumvented this obstacle [254]. Also, enhanced permeability due to aberrant vasculature in solid tumors and retention of ligand coated vectors around the receptors of tumor cells can increase chances for high probability of interaction with the cancer cells [43]. Thus, the nonviral vectors can acquire high gene transfer efficiency [43]. This concept was tested by preparing nonviral vector Tf-PEG-PEI-nanoparticles with plasmids packed inside an outer PEG-PEI layer coated with transferrin (Tf), an iron transporting protein [43, 254] that binds with Tf-receptors (Tf-R) with high affinity (depicted in the model in Figures 5 and 6). The Tf-R is present at much higher levels on the tumor cells [43] than on phenotypically normal epithelial cells. Association of transferrin with the Tf-PEG-PEI-nanoparticles significantly enhances transfection efficiency of shRNA generator-plasmids by promoting the internalization of Tf-PEG-PEI-nanoparticles in dividing and nondividing cells through receptor-mediated endocytosis [254]. The uptake of Tf-PEG-PEI-nanoparticles carrying multiple functional domains (surface shielding particles Tf-PEG-PEI, shRNA generator plasmids, tissue specific promoter driven-Cre-recombinase plasmids, and conditionally silenced plasmid) can overcome the intracellular barriers for successful delivery of the shRNA.

**Figure 7:**
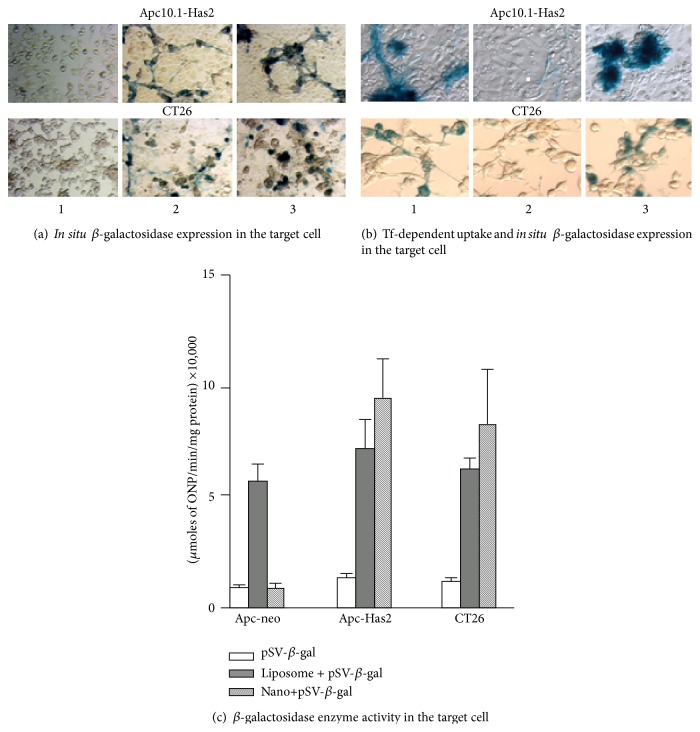
Uptake of pSV-*β*-galactosidase/Tf-PEG-PEI (nanoparticles) into target cell. Delivery of pSV-*β*-gal nanoparticles in colon cancer cells (adapted from [43]). (a)* In situ* *β*-galactosidase expression in the target cell. The* Apc*10.1-*HAS2* clone and the CT26 cells were treated for 48 h with the pSV-*β*-gal alone (*panel 1*), with the pSV-*β*-gal with liposome (*panel 2*), or with the pSV-*β*-gal/nanoparticles (35 nm average diameter, 8 *μ*g of pSV-*β*-gal/mL) (*panel 3*). The average size of the pSV-*β*-gal/nanoparticles is ~35 nm ± 20 nm. The transfected cells were fixed in 0.2% glutaraldehyde in PBS and washed twice in PBS. The cells were treated with a *β*-galactosidase staining solution and digitally photographed. (b) Transferrin-dependent uptake and* in situ* *β*-galactosidase expression in the target cell. The* Apc* 10.1-*HAS2* cells were transfected with the pSV-*β*-gal with liposome (*panel 1*), treated with Tf-R antibody and followed by transfection with the pSV-*β*-gal/nanoparticles (8 *μ*g pSV-*β*-gal/mL) (*panel 2*), or treated with the pSV-*β*-gal/nanoparticles (8 *μ*g pSV-*β*-gal/mL) alone (*panel 3*). The transfected cells were fixed in 0.2% glutaraldehyde in PBS and washed twice in PBS. *β*-galactosidase expressions in the cells were digitally photographed. (c) Cell-free extracts of parallel cultures were prepared in 10 mM CHAPS buffer and assayed for *β*-galactosidase activity using* o*-nitrophenyl *β*-D-galactopyranoside as substrate. The results are expressed as micromoles of* o*-nitrophenol formed per min/mg protein and represent ± S.D. of triplicate assays from the untransfected, liposome-transfected, or nanoparticles-treated cultures for each cell type.

**Figure 8:**
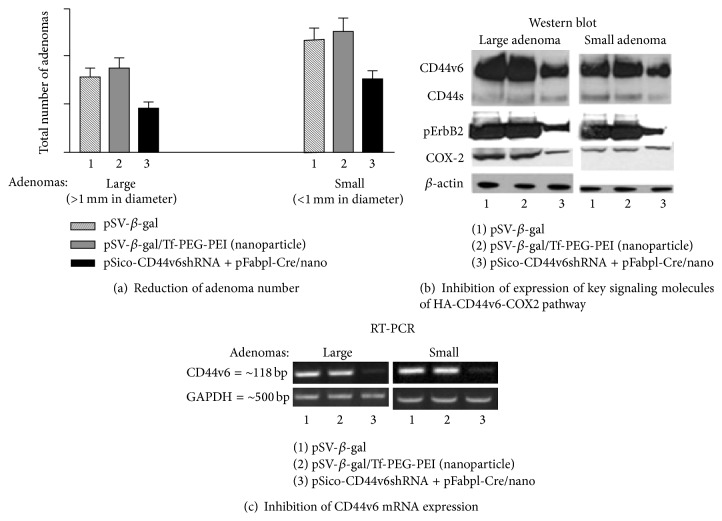
Systemic application of pSicoCD44v6shRNA plasmid in Apc Min/+ mice. Effect of plasmid/nanoparticle treatment on protein expression, RT-PCR analysis, and number of adenomas in Apc Min/+ mice (adapted from [43]). Thirty Apc Min/+ mice were randomly divided into three groups. Group 1 received pSV-*β*-galactosidase (100 *μ*g/100 *μ*L, intraperitoneally (i.p.)) alone, Group 2 received pSV-*β*-gal nanoparticles (100 *μ*g/100 *μ*L, i.p.) targeted to the Tf-R, and Group 3 received pSico-CD44v6shRNA (75 *μ*g) plus pFabpl-Cre (25 *μ*g)/nanoparticles i.p. every other day. 10 days after beginning treatment, the animals were sacrificed, and the large (>1 mm) and small (<1 mm) adenomas were counted (a). The tumor and adjacent normal tissues were subsequently processed for (b) western blots for CD44, pErbB2, TErbB2, COX-2, and *β*-actin and (c) RT-PCR analyses for CD44 variants from total RNA. Total ErbB2 remained unchanged in all the treatment groups (data not shown).

This shRNA plasmid delivery approach was tested for transfection of pSV-*β*-gal/Tf-PEG-PEI-nanoparticles in cellular models (Figure 7). Following this experiment, we successfully demonstrated that the CD44v6shRNA is localized into the colon tumor cells by an end-point assay of CD44v6 expression and by perturbation of HA-CD44v6 interaction as reflected in the reduction in the number of tumors [43] (Figure 8). The tissue specific shRNA delivery was made possible by the use of Cre-recombinase produced in response to a colon tissue specific promoter, which deletes the interruption between the U6 promoter and the CD44v6shRNA oligonucleotide. The newly developed cell-specific shRNA delivery approach by Misra et al. [43] confirmed that targeting the signaling pathways induced by HA-CD44v6 interaction inhibited distant colon tumor growth in Apc Min/+ mice. Our recent unpublished* in vivo* studies with the C57Bl/6 mice have now shown that systemic delivery of a mixture of two plasmids in Tf-PEG-PEI-nanoparticles (pARR_2_-Probasin-Cre/Tf-PEG-PEI-nanoparticles and floxed pSico-CD44v9shRNA/Tf-PEG-PEI-nanoparticles) can target both localized and metastatic prostate cancer cells. This novel approach opens up new ways to combat cancer and to understand tumorigenesis* in vivo* for the following reasons.

1. Cell specific shRNA to CD44 variant (CD44vshRNA) is released by applying a tissue specific promoter driven Cre-lox mechanism.
2. This shRNA silences the expression of the selected CD44 variant in the target tissue cancer cells.
3. This shRNA does not affect the normal target tissue cells, which rely on the standard CD44s and do not express the targeted CD44 variant and therefore are not affected by the plasmids.
4. The target CD44vshRNA will not be expressed in other types of cells because the tissue specific promoter only unlocks the Cre-recombinase in the targeted tissue cells thereby reducing potential side effects [43].
5. The Tf-PEG-PEI-nanoparticles that carry plasmids are biodegradable and cleared from the system.
6. This method inhibits the pathophysiological role of HA-CD44v interactions in cancer.
7. It can establish diagnostic markers for the targeted cancer, including CD44 variants, soluble CD44, and HA.
8. It can identify HA-CD44v interactions as innovative novel therapeutic targets against cancer progression.

Thus, the conditional suppression of gene expression by the use of an CD44vshRNA expressing plasmid holds potential promise for therapeutic approaches for silencing HA-CD44 variant signaling and downstream signaling pathways that promote disease causing genes [260].

## 5. Conclusions

**Figure 9:**
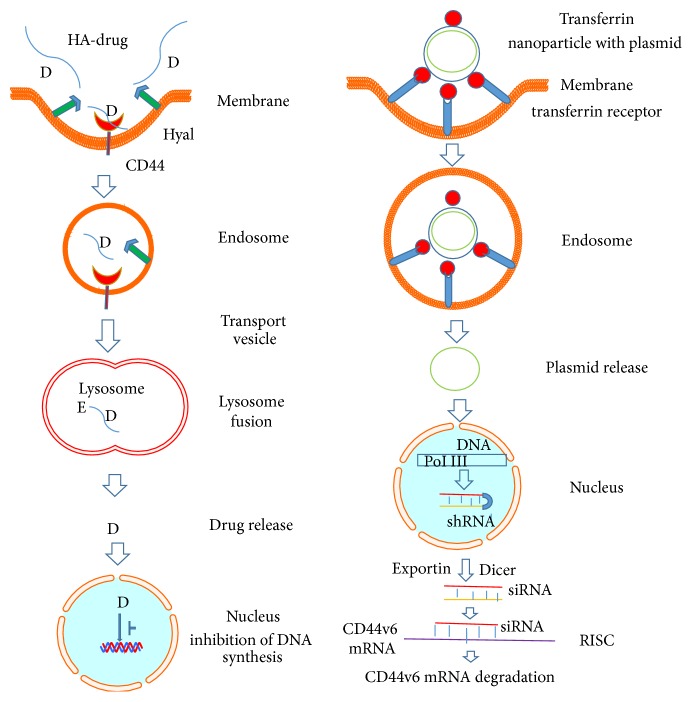
Exploitation of HA-CD44 interaction for anticancer therapy. Left panel represents the internalization of HA-drug conjugate that ultimately releases the drug that inhibits DNA synthesis of cancer cells. CD44 on the cell membrane binds the HA-drug conjugate and is internalized by endocytosis. The endosome formed is moved to the lysosome and fused. Here the HA in the conjugate is degraded first by hyaluronidase 1 (Hyal-1) into small HA oligosaccharides and next by lysosomal glycosidases to monosaccharides followed by release of the drug. The drug inhibits the DNA synthesis in the nucleus. Right panel exemplifies the steps that target the CD44v6mRNA in cancer cells by CD44v6shRNA. Plasmids producing CD44v6shRNA are coated with transferrin containing nanoparticles to target transferrin receptors of the cells. The particles are then internalized and form an endosome from which the plasmids are released to the nucleus where activation of DNA pol III occurs that results in CD44v6shRNA production. Through exportin, the newly produced CD44v6shRNA come out into cytoplasm where it is converted into CD44v6siRNA by dicer enzyme. One of the strands of siRNA will bind to CD44v6mRNA and forms RNA-induced silencing complex (RISC) which is ultimately degraded.

Among various GAGs and PGs, much research has demonstrated the ability of HA to target cancer cells overexpressing the HA receptor CD44, in particular its variants, and that HA interaction with CD44v augments cancer pathobiology (Figure 9). Thus, interference with the function of HA-CD44 can inhibit the malignant process at multiple stages. This can be accomplished by perturbation of HA-CD44 signaling pathways and disruption of the HA matrix with hyaluronidases to facilitate passive carrier uptake, targeting the HA tumor matrix and providing sustained source of drug to the tumor site or by targeting CD44 receptor by CD44 blocking antibody or tissue specific targeting of specific variants of CD44 that are overexpressed in tumors. Cancer is a disease of the organism and is the subject of intense research around the world, but many questions about how the disease works remain unanswered. However, the multifaceted functions of GAGs and PGs require careful, context-dependent therapeutic applications because they have dual functions; and targeting ECM GAGs and PGs may promote escape of tumor cells from the primary tumor by inhibiting cancer cell attachment and increasing distant metastatic migration of tumor cells. Alternative viable and beneficial approaches are targeting the tumor ECM to disrupt HA-CD44v signaling pathways, keeping the function of CD44s intact. Thus, our validated tumor specific delivery of CD44 variant-shRNA has considerable advantage versus other therapeutic strategies. First, this technique avoids multiple chemical steps to prepare HA conjugated cytotoxic drugs and conjugation to nanocarriers. Second, it abolishes the CD44v variants in the cancer cells only. Third, a number of cell types in normal tissues that express CD44s or the hematopoietic form will not be affected because these are not activated. Fourth, although inflammation-associated cancers accumulate activated immune cells with upregulated transferrin receptors and CD44 variants and may take up the Tf-PEG-PEI-nanoparticles, there will be no deletion of CD44 variant because the promoter is not lymphocyte specific. To target activated lymphocytes, specific lymphocyte promoter driven-Cre plasmids would have to be used. Fifth, accumulation of antibody in nontumor areas is a major limitation of anti-CD44 antibody therapy. Experiments so far have not shown any such effect in shRNA delivery. The HA-CD44 interaction system is illustrated in Figure 9 where we specify alternatives for cancer therapeutical aspects (discussed in this review) that specifically perturb HA-CD44 signaling pathways.

In conclusion, CD44 is the most prevalent cell surface marker of cancer stem cells (CSCs) [230, 262–264]. CD44 transcripts undergo complex alternative splicing of CD44 precursor mRNA under the influence of epithelial splicing regulatory protein 1 (ESRP1) [18, 70], giving rise to functionally different CD44 variant isoforms (CD44*v*1–*v*10), encoded by one single gene. Importantly, ablation of CD44v6 or ESRP1 with our CD44vshRNA/Tf-PEG-PEI-nanoparticles or ESRP1shRNA/Tf-PEG-PEI-nanoparticles will have important potential to reduce tumor growth that involves overexpression of CD44v or ESRP1, indicating the importance of tissue specific delivery of shRNA/nanoparticle technology.

## Figures and Tables

**Table 1:** Noncancer drug delivery applications of GAG delivery systems.

Application	Components	Drug	References
Transcutaneous delivery	Hyaluronic acid microneedles	Insulin	[134]

Transcutaneous immunization	Hyaluronic acid microneedles	Tetanus, Diphtheria, Malaria, and Influenza	[141, 142]

Topical delivery on burn sites	Hyaluronic acid	Anti-IL-6-hyaluronic acid conjugate. Anti-TNF-*α*-hyaluronic acid conjugate	[151]

Ocular delivery	Hyaluronic acid	Pilocarpine, timolol against glaucoma, tropicamide for dilation of pupil, tobramycin against bacterial infection of eye	[159–161, 163] [162, 163]

Oral delivery	Heparin is conjugated with taurocholic acid (TCA)	Docetaxel (DTX)	[86, 87]

Intra-articular injection	LMW heparin-N,N,N trimethylchitosan chloride (TMC)-nanoparticles, Heparin-Chitosan-nanoparticles hyaluronic acid-heparin hydrogel	Heparin, VEGF, and bFGF Growth factor, for example, BMP2	[89] [91]

Intracellular delivery	Cell penetrating peptide (CPP)	Peptides, oligonucleotides, siRNAs, and liposomes	[98–103, 107]

**Table 2:** Cancer drug delivery applications of GAG/HA delivery systems.

Application	Components	Drug	References
Antimitotic delivery in bladder carcinoma cells	Hyaluronic acid (HA) 10–12 KDa	Paclitaxel	[176, 177, 181]

Anti-DNA winding delivery In CD44 overexpressing colon, breast, esophageal, and ovarian cancer	Hyaluronic acid 200 KDa	Irinotecan	[178, 182]

HA-encapsulated drug delivery for metastatic breast, prostate and colon cancer	Hyaluronic acid ~750 KDa	5-FU, doxorubicin	[185, 186]

Localized delivery in bone disease in cancer	Hyaluronic acid	Bisphosphonate	[187]

Nanoparticle delivery in colon cancer	Chitosan-hyaluronic acid	5-FU	[190, 191]

Peritumoral delivery of nanovectors in head/neck cancer (HNSCC)	Lipid-Hyaluronic acid (1.2–5 MDa)	Mitomycin C	[192]
	Hyaluronic acid	Cisplatin	[183]

Intracellular delivery of polymeric micelles for CD44 and folate receptor overexpressing breast and lung cancer and in cancer stem cells	Folic acid conjugated HA-C18 micelles Folate linked CS on surfaces of Pluronic 127 nanogels	Paclitaxol, doxorubicin, salinomycin, and doxorubicin	[199–201] [123]

Intracellular delivery of CD44 overexpressing cancer (breast, colon, and HNSCC)	HA-nanocarrier	Epirubicin, doxorubicin	[50, 176, 196]
		Paclitaxel, mitomycin C, siRNAs	[202]

Intracellular delivery of nanogels in breast cancer	Doxorubicin cores coated with dextran sulfate (DS 435)-nanoparticles	Doxorubicin	[124]

**Table 3:** Therapeutic applications of disrupting the HA/GAG biological systems.

Application	Components	Drug	References
Phase I trial for maximum tolerated dose and safety profile in intraperitoneal infusion in ovarian, breast, colon, stomach, and bladder cancer	Hyaluronic acid (HA) 10–12 KDa	Paclitaxel	[177]

*In vivo* cytotoxicity in peritoneal colorectal cancer	Hyaluronic acid ~200 KDa	Irrinotecan	[182]

HA-encapsulated drug delivery for metastatic breast, prostate, and colon cancer	Hyaluronic acid ~750 KDa	5-FU, Doxorubicin	[185, 186]

Localized delivery in bone disease in cancer	Hyaluronic acid	Bisphosphonate	[187]

Nanoparticle delivery in colon cancer	Chitosan-hyaluronic acid	5-FU	[190, 191]

Peritumoral delivery of nanovectors in head/neck squamas cell cancer (HNSCC)	Lipid-hyaluronic acid (1.2–5 MDa)	Mitomycin C	[192]
	Hyaluronic acid	Cisplatin	[183]

Targeting with anti-CD44 antibodies in cancers including myeloid leukemia and HNSCC	Anti-CD44 antibody (Ab), humanized anti-CD44v6 monoclonal antibody (HAMA) labelled with technetium-99m	Anti-CD44Ab, Anti-CD44v6Ab	[231, 242–247]

Targeting *in vitro* breast cancer cells and xenograft lung tumor by interrupting HA-CD44 interaction	Small HA-oligosaccharides	6–18 saccharide units (oHAs)	[11, 13, 41, 248, 249]

Targeting enzymatic degradation of HA in EMT-6 breast cancer cells, bladder cancer cells	bovine testicular, bacterial hyaluronidases, HYAL1-*v*1		[250, 251] [252]

Targeting leukemia cell by nonviral vectors in leukemia K562 cells	Transferrin-PEG-test luciferase plasmid-nanoparticle	test luciferase plasmid	[253, 254]

*In vivo* targeting CD44v6 by tissue specific deletion of CD44 variant in intestine/colon tumor in Apc Min/+ mice	pSico-CD44v6shRNA plus pFabpl-Cre	CD44v6shRNA	[18, 43, 44, 255]

## Abbreviations

GAG: Glycosaminoglycan
PG: Proteoglycan
HA: Hyaluronan
ECM: Extracellular matrix
HAS: Hyaluronan synthase
HYALs: Hyaluronidases
shRNA: Short hairpin RNA
Tf: Transferrin
TfR: Transferrin receptor
Plasmids/Tf-PEG-PEI: Plasmids/Transferrin-polyethylene glycol-polyethylene imine.

## References

[1] Wolf K., Wu Y. I., Liu Y. (2007). Multi-step pericellular proteolysis controls the transition from individual to collective cancer cell invasion. *Nature Cell Biology*.

[2] Sabeh F., Shimizu-Hirota R., Weiss S. J. (2009). Protease-dependent versus-independent cancer cell invasion programs: three-dimensional amoeboid movement revisited. *The Journal of Cell Biology*.

[3] Hardingham T. E., Fosang A. J. (1992). Proteoglycans: many forms and many functions. *The FASEB Journal*.

[4] Mulloy B., Rider C. C. (2006). Cytokines and proteoglycans: an introductory overview. *Biochemical Society Transactions*.

[5] Bishop J. R., Schuksz M., Esko J. D. (2007). Heparan sulphate proteoglycans fine-tune mammalian physiology. *Nature*.

[6] Mythreye K., Blobe G. C. (2009). Proteoglycan signaling co-receptors: roles in cell adhesion, migration and invasion. *Cellular Signalling*.

[7] Bernfield M., Götte M., Park P. W. (1999). Functions of cell surface heparan sulfate proteoglycans. *Annual Review of Biochemistry*.

[8] Yang J., Price M. A., Neudauer C. L. (2004). Melanoma chondroitin sulfate proteoglycan enhances FAK and ERK activation by distinct mechanisms. *Journal of Cell Biology*.

[9] Reed C. C., Waterhouse A., Kirby S. (2005). Decorin prevents metastatic spreading of breast cancer. *Oncogene*.

[10] Ghatak S., Misra S., Toole B. P. (2005). Hyaluronan constitutively regulates ErbB2 phosphorylation and signaling complex formation in carcinoma cells. *Journal of Biological Chemistry*.

[11] Misra S., Ghatak S., Toole B. P. (2005). Regulation of MDR1 expression and drug resistance by a positive feedback loop involving hyaluronan, phosphoinositide 3-kinase, and ErbB2. *The Journal of Biological Chemistry*.

[12] Misra S., Obeid L. M., Hannun Y. A. (2008). Hyaluronan constitutively regulates activation of COX-2-mediated cell survival activity in intestinal epithelial and colon carcinoma cells. *Journal of Biological Chemistry*.

[13] Misra S., Toole B. P., Ghatak S. (2006). Hyaluronan constitutively regulates activation of multiple receptor tyrosine kinases in epithelial and carcinoma cells. *Journal of Biological Chemistry*.

[14] Simpson M. A., Wilson C. M., McCarthy J. B. (2002). Inhibition of prostate tumor cell hyaluronan synthesis impairs subcutaneous growth and vascularization in immunocompromised mice. *The American Journal of Pathology*.

[15] Udabage L., Brownlee G. R., Waltham M. (2005). Antisense-mediated suppression of hyaluronan synthase 2 inhibits the tumorigenesis and progression of breast cancer. *Cancer Research*.

[16] Camenisch T. D., Schroeder J. A., Bradley J., Klewer S. E., McDonald J. A. (2002). Heart-valve mesenchyme formation is dependent on hyaluronan-augmented activation of ErbB2-ErbB3 receptors. *Nature Medicine*.

[17] Camenisch T. D., Spicer A. P., Brehm-Gibson T. (2000). Disruption of hyaluronan synthase-2 abrogates normal cardiac morphogenesis and hyaluronan-mediated transformation of epithelium to mesenchyme. *Journal of Clinical Investigation*.

[18] Misra S., Heldin P., Hascall V. C. (2011). Hyaluronan-CD44 interactions as potential targets for cancer therapy. *FEBS Journal*.

[19] Misra S., Hascall V., Karamanos N., Markwald R. A., Ghatak S. (2012). *Targeting Tumor Microenvironment in Cancer Progression*.

[20] Hascall V. C., Laurent T. (1997). *Hyaluronan: Structure and Physical Properties*.

[21] Hascall V. C., Majors A. K., De La Motte C. A. (2004). Intracellular hyaluronan: a new frontier for inflammation?. *Biochimica et Biophysica Acta—General Subjects*.

[22] Spicer A. P., Tien J. Y. L. (2004). Hyaluronan and morphogenesis. *Birth Defects Research Part C: Embryo Today*.

[23] Heldin P., Pertoft H. (1993). Synthesis and assembly of the hyaluronan-containing coats around normal human mesothelial cells. *Experimental Cell Research*.

[24] Evanko S. P., Parks T., Wight T. N. (2004). Intracellular hyaluronan in arterial smooth muscle cells: association with microtubules, RHAMM, and the mitotic spindle. *Journal of Histochemistry & Cytochemistry*.

[25] Toole B. P. (2004). Hyaluronan: from extracellular glue to pericellular cue. *Nature Reviews Cancer*.

[26] Meyer K., Palmer J. W. (1934). The polysaccharide of the vitreous humor. *Journal of Biological Chemistry*.

[27] Olczyk P., Komosińska-Vassev K., Winsz-Szczotka K., Kuźnik-Trocha K., Olczyk K. (2008). Hyaluronan: structure, metabolism, functions, and role in wound healing. *Postępy Higieny i Medycyny Doświadczalnej*.

[28] Gandhi N. S., Mancera R. L. (2008). The structure of glycosaminoglycans and their interactions with proteins. *Chemical Biology and Drug Design*.

[29] Laurent T. C., Fraser J. R. E. (1992). Hyaluronan. *The FASEB Journal*.

[30] Chen W. Y. J., Abatangelo G. (1999). Functions of hyaluronan in wound repair. *Wound Repair and Regeneration*.

[31] Weigel P. H., DeAngelis P. L. (2007). Hyaluronan synthases: a decade-plus of novel glycosyltransferases. *The Journal of Biological Chemistry*.

[32] Stern R., Jedrzejas M. J. (2006). Hyaluronidases: their genomics, structures, and mechanisms of action. *Chemical Reviews*.

[33] Banerji S., Ni J., Wang S. X. (1999). LYVE-1, a new homologue of the CD44 glycoprotein, is a lymph-specific receptor for hyaluronan. *Journal of Cell Biology*.

[34] Zhou B., Weigel J. A., Fauss L., Weigel P. H. (2000). Identification of the hyaluronan receptor for endocytosis (HARE). *The Journal of Biological Chemistry*.

[35] Toole B. P., Zoltan-Jones A., Misra S., Ghatak S. (2005). Hyaluronan: a critical component of epithelial-mesenchymal and epithelial-carcinoma transitions. *Cells Tissues Organs*.

[36] Pilarski L. M., Masellis-Smith A., Belch A. R., Yang B., Savani R. C., Turley E. A. (1994). RHAMM, a receptor for hyaluronan-mediated motility, on normal human lymphocytes, thymocytes and malignant B cells: a mediator in B cell malignancy?. *Leukemia and Lymphoma*.

[37] Wang Y., Du H., Zhai G. (2014). Recent advances in active hepatic targeting drug delivery system. *Current Drug Targets*.

[38] Ghatak S., Bogatkevich G. S., Atnelishvilis I. (2014). Overexpression of c-Met and CD44v6 receptors contributes to autocrine TGF-*β*1 signaling in interstitial lung disease. *Journal of Biological Chemistry*.

[39] Ghatak S., Hascall V. C., Markwald R. R., Misra S. (2010). Stromal hyaluronan interaction with epithelial CD44 variants promotes prostate cancer invasiveness by augmenting expression and function of hepatocyte growth factor and androgen receptor. *Journal of Biological Chemistry*.

[40] Ghatak S., Misra S., Norris R. A. (2014). Periostin induces intracellular cross-talk between kinases and hyaluronan in atrioventricular valvulogenesis. *The Journal of Biological Chemistry*.

[41] Ghatak S., Misra S., Toole B. P. (2002). Hyaluronan oligosaccharides inhibit anchorage-independent growth of tumor cells by suppressing the phosphoinositide 3-kinase/Akt cell survival pathway. *Journal of Biological Chemistry*.

[42] Misra S., Hascall V. C., Berger F. G., Markwald R. R., Ghatak S. (2008). Hyaluronan, CD44, and cyclooxygenase-2 in colon cancer. *Connective Tissue Research*.

[43] Misra S., Hascall V. C., De Giovanni C., Markwald R. R., Ghatak S. (2009). Delivery of CD44 shRNA/nanoparticles within cancer cells. Perturbation of hyaluronan/CD44v6 interactions and reduction in adenoma growth in Apc Min/+mice. *Journal of Biological Chemistry*.

[44] Misra S., Hascall V. C., Karamanos N. K., Markwald R. R., Ghatak S. (2012). *Delivery Systems Targeting Cancer at the Level of ECM*.

[45] Bourguignon L. Y. W., Peyrollier K., Xia W., Gilad E. (2008). Hyaluronan-CD44 interaction activates stem cell marker Nanog, Stat-3-mediated MDR1 gene expression, and ankyrin-regulated multidrug efflux in breast and ovarian tumor cells. *The Journal of Biological Chemistry*.

[46] Bourguignon L. Y. W., Singleton P. A., Zhu H., Diedrich F. (2003). Hyaluronan-mediated CD44 interaction with RhoGEF and Rho kinase promotes Grb2-associated binder-1 phosphorylation and phosphatidylinositol 3-kinase signaling leading to cytokine (macrophage-colony stimulating factor) production and breast tumor progression. *Journal of Biological Chemistry*.

[47] Bourguignon L. Y. W., Peyrollier K., Gilad E., Brightman A. (2007). Hyaluronan-CD44 interaction with neural Wiskott-Aldrich syndrome protein (N-WASP) promotes actin polymerization and ErbB2 activation leading to beta-catenin nuclear translocation, transcriptional up-regulation, and cell migration in ovarian tumor cells. *The Journal of Biological Chemistry*.

[48] Naor D., Nedvetzki S., Golan I., Melnik L., Faitelson Y. (2002). CD44 in cancer. *Critical Reviews in Clinical Laboratory Sciences*.

[49] Bourguignon L. Y. W., Zhu H., Shao L., Chen Y. W. (2000). CD44 interaction with tiam1 promotes Rac1 signaling and hyaluronic acid- mediated breast tumor cell migration. *The Journal of Biological Chemistry*.

[50] Akima K., Ito H., Iwata Y. (1996). Evaluation of antitumor activities of hyaluronate binding antitumor drugs: synthesis, characterization and antitumor activity. *Journal of Drug Targeting*.

[51] Park J. I., Cao L., Platt V. M. (2009). Antitumor therapy mediated by 5-fluorocytosine and a recombinant fusion protein containing TSG-6 hyaluronan binding domain and yeast cytosine deaminase. *Molecular Pharmaceutics*.

[52] Sy M.-S., Guo Y.-J., Stamenkovic I. (1992). Inhibition of tumor growth in vivo with a soluble CD44-immunoglobulin fusion protein. *The Journal of Experimental Medicine*.

[53] Lokeshwar V. B., Lopez L. E., Munoz D. (2010). Antitumor activity of hyaluronic acid synthesis inhibitor 4-methylumbelliferone in prostate cancer cells. *Cancer Research*.

[54] Arai E., Nishida Y., Wasa J. (2011). Inhibition of hyaluronan retention by 4-methylumbelliferone suppresses osteosarcoma cells *in vitro* and lung metastasis *in vivo*. *British Journal of Cancer*.

[55] Nakazawa H., Yoshihara S., Kudo D. (2006). 4-methylumbelliferone, a hyaluronan synthase suppressor, enhances the anticancer activity of gemcitabine in human pancreatic cancer cells. *Cancer Chemotherapy and Pharmacology*.

[56] Klocker J., Sabitzer H., Raunik W., Wieser S., Schumer J. (1998). Hyaluronidase as additive to induction chemotherapy in advanced squamous cell carcinoma of the head and neck. *Cancer Letters*.

[57] Pillwein K., Fuiko R., Slavc I. (1998). Hyaluronidase additional to standard chemotherapy improves outcome for children with malignant brain tumors. *Cancer Letters*.

[58] Whatcott C. J., Han H., Posner R. G., Hostetter G., Von Hoff D. D. (2011). Targeting the tumor microenvironment in cancer: why hyaluronidase deserves a second look. *Cancer Discovery*.

[59] Fraser J. R. E., Kimpton W. G., Laurent T. C., Cahill R. N. P., Vakakis N. (1988). Uptake and degradation of hyaluronan in lymphatic tissue. *Biochemical Journal*.

[60] Fraser J. R., Laurent T. C. (1989). Turnover and metabolism of hyaluronan. *Ciba Foundation Symposium*.

[61] Ostgaard G., Reed R. K. (1993). Hyaluronan turnover in the rat small intestine. *Acta Physiologica Scandinavica*.

[62] Ostgaard G., Reed R. K. (1993). Intravenous saline infusion in rat increases hyaluronan efflux in intestinal lymph by increasing lymph flow. *Acta Physiologica Scandinavica*.

[63] Ostgaard G., Reed R. K. (1994). Increased lymphatic hyaluronan output and preserved hyaluronan content of the rat small intestine in prolonged hypoproteinaemia. *Acta Physiologica Scandinavica*.

[64] Dahl L. B., Laurent T. C., Smedsrød B. (1988). Preparation of biologically intact radioiodinated hyaluronan of high specific radioactivity: coupling of ^125^I-tyramine-cellobiose to amino groups after partial N-deacetylation. *Analytical Biochemistry*.

[65] Fraser J. R. E., Laurent T. C., Pertoft H., Baxter E. (1981). Plasma clearance, tissue distribution and metabolism of hyaluronic acid injected intravenously in the rabbit. *Biochemical Journal*.

[66] Feusi E., Sun L., Sibalic A., Beck-Schimmer B., Oertli B., Wüthrich R. P. (1999). Enhanced hyaluronan synthesis in the MRL-Fas^lpr^ kidney: role of cytokines. *Nephron*.

[67] Sun L.-K., Feusi E., Sibalic A., Beck-Schimmer B., Wüthrich R. P. (1998). Expression profile of hyaluronidase mRNA transcripts in the kidney and in renal cells. *Kidney and Blood Pressure Research*.

[68] Sibalic V., Fan X., Loffing J., Wüthrich R. P. (1997). Upregulated renal tubular CD44, hyaluronan, and osteopontin in kdkd mice with interstitial nephritis. *Nephrology Dialysis Transplantation*.

[69] Benz P. S., Fan X., Wüthrich R. P. (1996). Enhanced tubular epithelial CD44 expression in MRL-lpr lupus nephritis. *Kidney International*.

[70] Ponta H., Sherman L., Herrlich P. A. (2003). CD44: from adhesion molecules to signalling regulators. *Nature Reviews Molecular Cell Biology*.

[71] van der Voort R., Taher T. E. I., Wielenga V. J. M. (1999). Heparan sulfate-modified CD44 promotes hepatocyte growth factor/scatter factor-induced signal transduction through the receptor tyro sine kinase c- Met. *The Journal of Biological Chemistry*.

[72] Skelton T. P., Zeng C., Nocks A., Stamenkovic I. (1998). Glycosylation provides both stimulatory and inhibitory effects on cell surface and soluble CD44 binding to hyaluronan. *Journal of Cell Biology*.

[73] Naor D., Wallach-Dayan S. B., Zahalka M. A., Sionov R. V. (2008). Involvement of CD44, a molecule with a thousand faces, in cancer dissemination. *Seminars in Cancer Biology*.

[74] Echiburú-Chau C., Roy D., Calaf G. M. (2011). Metastatic suppressor CD44 is related with oxidative stress in breast cancer cell lines. *International Journal of Oncology*.

[75] Louderbough J. M. V., Brown J. A., Nagle R. B., Schroeder J. A. (2011). CD44 promotes epithelial mammary gland development and exhibits altered localization during cancer progression. *Genes and Cancer*.

[76] Louderbough J. M. V., Schroeder J. A. (2011). Understanding the dual nature of CD44 in breast cancer progression. *Molecular Cancer Research*.

[77] Vinogradov S. V., Bronich T. K., Kabanov A. V. (2002). Nanosized cationic hydrogels for drug delivery: preparation, properties and interactions with cells. *Advanced Drug Delivery Reviews*.

[78] Qiu L. Y., Bae Y. H. (2006). Polymer architecture and drug delivery. *Pharmaceutical Research*.

[79] Svenson S., Tomalia D. A. (2005). Dendrimers in biomedical applications—reflections on the field. *Advanced Drug Delivery Reviews*.

[80] Häcker U., Nybakken K., Perrimon N. (2005). Heparan sulphate proteoglycans: the sweet side of development. *Nature Reviews Molecular Cell Biology*.

[81] Jain R. K., Munn L. L., Fukumura D. (2002). Dissecting tumour pathophysiology using intravital microscopy. *Nature Reviews Cancer*.

[82] Chauhan V. P., Stylianopoulos T., Boucher Y., Jain R. K. (2011). Delivery of molecular and nanoscale medicine to tumors: transport barriers and strategies. *Annual Review of Chemical and Biomolecular Engineering*.

[83] Linhardt R. J. (2003). 2003 Claude S. Hudson award address in carbohydrate chemistry. Heparin: structure and activity. *Journal of Medicinal Chemistry*.

[84] Clemons K. V., Ranney D. F., Stevens D. A. (2001). A novel heparin-coated hydrophilic preparation of amphotericin B hydrosomes. *Current Opinion in Investigational Drugs*.

[85] Lee D. Y., Khatun Z., Lee J.-H., Lee Y.-K., In I. (2011). Blood compatible graphene/heparin conjugate through noncovalent chemistry. *Biomacromolecules*.

[86] Khatun Z., Nurunnabi M., Reeck G. R., Cho K. J., Lee Y.-K. (2013). Oral delivery of taurocholic acid linked heparin-docetaxel conjugates for cancer therapy. *Journal of Controlled Release*.

[87] Khatun Z., Nurunnabi M., Cho K. J., Byun Y., Bae Y. H., Lee Y.-K. (2014). Oral absorption mechanism and anti-angiogenesis effect of taurocholic acid-linked heparin-docetaxel conjugates. *Journal of Controlled Release*.

[88] Tan H., Shen Q., Jia X., Yuan Z., Xiong D. (2012). Injectable nanohybrid scaffold for biopharmaceuticals delivery and soft tissue engineering. *Macromolecular Rapid Communications*.

[89] Tang D.-W., Yu S.-H., Ho Y.-C., Mi F.-L., Kuo P.-L., Sung H.-W. (2010). Heparinized chitosan/poly(*γ*-glutamic acid) nanoparticles for multi-functional delivery of fibroblast growth factor and heparin. *Biomaterials*.

[90] Xu X., Jha A. K., Duncan R. L., Jia X. (2011). Heparin-decorated, hyaluronic acid-based hydrogel particles for the controlled release of bone morphogenetic protein 2. *Acta Biomaterialia*.

[91] Srinivasan P. P., McCoy S. Y., Jha A. K. (2012). Injectable perlecan domain 1-hyaluronan microgels potentiate the cartilage repair effect of BMP2 in a murine model of early osteoarthritis. *Biomedical Materials*.

[92] Poole A. R. (1986). Proteoglycans in health and disease: structures and functions. *Biochemical Journal*.

[93] Prydz K., Dalen K. T. (2000). Synthesis and sorting of proteoglycans. *Journal of Cell Science*.

[94] Bernfield M., Kokenyesi R., Kato M. (1992). Biology of the syndecans: a family of transmembrane heparan sulfate proteoglycans. *Annual Review of Cell Biology*.

[95] Christianson H. C., Belting M. (2014). Heparan sulfate proteoglycan as a cell-surface endocytosis receptor. *Matrix Biology*.

[96] Guo Y.-M., Liu M., Yang J.-L. (2007). Intercellular imaging by a polyarginine derived cell penetrating peptide labeled magnetic resonance contrast agent, diethylenetriamine pentaacetic acid gadolinium. *Chinese Medical Journal*.

[97] Letoha T., Keller-Pintér A., Kusz E. (2010). Cell-penetrating peptide exploited syndecans. *Biochimica et Biophysica Acta*.

[98] Wang J., Lu Z., Wientjes M. G., Au J. L.-S. (2010). Delivery of siRNA therapeutics: barriers and carriers. *AAPS Journal*.

[99] Said Hassane F., Saleh A. F., Abes R., Gait M. J., Lebleu B. (2010). Cell penetrating peptides: overview and applications to the delivery of oligonucleotides. *Cellular and Molecular Life Sciences*.

[100] Brooks N. A., Pouniotis D. S., Sheng K. C., Apostolopoulos V., Pietersz G. A. (2010). A membrane penetrating multiple antigen peptide (MAP) incorporating ovalbumin CD8 epitope induces potent immune responses in mice. *Biochimica et Biophysica Acta—Biomembranes*.

[101] Brooks N. A., Pouniotis D. S., Tang C.-K., Apostolopoulos V., Pietersz G. A. (2010). Cell-penetrating peptides: application in vaccine delivery. *Biochimica et Biophysica Acta*.

[102] Fretz M. M., Storm G. (2010). TAT-peptide modified liposomes: preparation, characterization, and cellular interaction. *Methods in Molecular Biology*.

[103] Patel L. N., Wang J., Kim K.-J., Borok Z., Crandall E. D., Shen W.-C. (2009). Conjugation with cationic cell-penetrating peptide increases pulmonary absorption of insulin. *Molecular Pharmaceutics*.

[104] Kleeff J., Ishiwata T., Kumbasar A. (1998). The cell-surface heparan sulfate proteoglycan glypican-1 regulates growth factor action in pancreatic carcinoma cells and is overexpressed in human pancreatic cancer. *Journal of Clinical Investigation*.

[105] Sharma B., Handler M., Eichstetter I., Whitelock J. M., Nugent M. A., Iozzo R. V. (1998). Antisense targeting of perlecan blocks tumor growth and angiogenesis in vivo. *The Journal of Clinical Investigation*.

[106] Grubb J. H., Vogler C., Sly W. S. (2010). New strategies for enzyme replacement therapy for lysosomal storage diseases. *Rejuvenation Research*.

[107] Wraith J. E. (2002). Lysosomal disorders. *Seminars in Neonatology*.

[108] Hamada K., Yoshihara C., Ito T. (2012). Antitumor effect of chondroitin sulfate-coated ternary granulocyte macrophage-colony-stimulating factor plasmid complex for ovarian cancer. *Journal of Gene Medicine*.

[109] Liu Z., Jiao Y., Wang Y., Zhou C., Zhang Z. (2008). Polysaccharides-based nanoparticles as drug delivery systems. *Advanced Drug Delivery Reviews*.

[110] Sinha V. R., Kumria R. (2001). Polysaccharides in colon-specific drug delivery. *International Journal of Pharmaceutics*.

[111] Soppimath K. S., Aminabhavi T. M., Kulkarni A. R., Rudzinski W. E. (2001). Biodegradable polymeric nanoparticles as drug delivery devices. *Journal of Controlled Release*.

[112] Mitra S., Gaur U., Ghosh P. C., Maitra A. N. (2001). Tumour targeted delivery of encapsulated dextran-doxorubicin conjugate using chitosan nanoparticles as carrier. *Journal of Controlled Release*.

[113] Lee C.-T., Huang C.-P., Lee Y.-D. (2006). Preparation of amphiphilic poly(L-lactide)-graft-chondroitin sulfate copolymer self-aggregates and its aggregation behavior. *Biomacromolecules*.

[114] Mocanu G., Mihai D., Picton L., LeCerf D., Muller G. (2002). Associative pullulan gels and their interaction with biological active substances. *Journal of Controlled Release*.

[115] Campoli M. R., Chang C.-C., Kageshita T., Wang X., McCarthy J. B., Ferrone S. (2004). Human high molecular weight-melanoma-associated antigen (HMW-MAA): a melanoma cell surface chondroitin sulfate proteoglycan (MSCP) with biological and clinical significance. *Critical Reviews in Immunology*.

[116] Yang J., Price M. A., Gui Y. L. (2009). Melanoma proteoglycan modifies gene expression to stimulate tumor cell motility, growth, and epithelial-to-mesenchymal transition. *Cancer Research*.

[117] Iida J., Wilhelmson K. L., Ng J. (2007). Cell surface chondroitin sulfate glycosaminoglycan in melanoma: role in the activation of pro-MMP-2 (pro-gelatinase A). *Biochemical Journal*.

[118] Chang C.-C., Campoli M., Luo W., Zhao W., Zaenker K. S., Ferrone S. (2004). Immunotherapy of melanoma targeting human high molecular weight melanoma-associated antigen: potential role of nonimmunological mechanisms. *Annals of the New York Academy of Sciences*.

[119] Schwenkert M., Birkholz K., Schwemmlein M. (2008). A single chain immunotoxin, targeting the melanoma-associated chondroitin sulfate proteoglycan, is a potent inducer of apoptosis in cultured human melanoma cells. *Melanoma Research*.

[120] Xi J., Zhou L., Fei Y. (2012). Preparation of chondroitin sulfate nanocapsules for use as carries by the interfacial polymerization method. *International Journal of Biological Macromolecules*.

[121] Xi J., Qin J., Fan L. (2012). Chondroitin sulfate functionalized mesostructured silica nanoparticles as biocompatible carriers for drug delivery. *International Journal of Nanomedicine*.

[122] Park W., Park S.-J., Na K. (2010). Potential of self-organizing nanogel with acetylated chondroitin sulfate as an anti-cancer drug carrier. *Colloids and Surfaces B: Biointerfaces*.

[123] Huang S.-J., Sun S.-L., Feng T.-H., Sung K.-H., Lui W.-L., Wang L.-F. (2009). Folate-mediated chondroitin sulfate-Pluronic 127 nanogels as a drug carrier. *European Journal of Pharmaceutical Sciences*.

[124] Mascellani G., Liverani L., Bianchini P. (1993). Structure and contribution to the heparin cofactor II-mediated inhibition of thrombin of naturally oversulphated sequences of dermatan sulphate. *Biochemical Journal*.

[125] Meyer K., Linker A., Davidson E. A., Weissmann B. (1953). The mucopolysaccharides of bovine cornea. *The Journal of Biological Chemistry*.

[126] Hassell J. R., Newsome D. A., Krachmer J. H., Rodrigues M. M. (1980). Macular corneal dystrophy: failure to synthesize a mature keratan sulfate proteoglycan. *Proceedings of the National Academy of Sciences of the United States of America*.

[127] Li T. C., Aplin J. D., Warren A., Graham R. A., Dockery P., Cooke I. D. (1994). Endometrial responses to three different progestins in artificial cycles: a prospective, crossover study. *Fertility and Sterility*.

[128] Graham R. A., Li T. C., Cooke I. D., Aplin J. D. (1994). Keratan sulphate as a secretory product of human endometrium: cyclic expression in normal women. *Human Reproduction*.

[129] Funderburgh J. L. (2000). Keratan sulfate: structure, biosynthesis, and function. *Glycobiology*.

[130] Funderburgh J. L., Funderburgh M. L., Mann M. M., Conrad G. W. (1991). Physical and biological properties of keratan sulphate proteoglycan. *Biochemical Society Transactions*.

[131] Hascall V. C. (1982). Structure and biosynthesis of proteoglycans with keratan sulfate. *Progress in Clinical and Biological Research*.

[132] Greiling H. (1994). Structure and biological functions of keratan sulfate proteoglycans. *EXS*.

[133] Tomatsu S., Montão A. M., Dung V. C. (2010). Enhancement of drug delivery: enzyme-replacement therapy for murine Morquio A syndrome. *Molecular Therapy*.

[134] Liu S., Jin M.-N., Quan Y.-S. (2012). The development and characteristics of novel microneedle arrays fabricated from hyaluronic acid, and their application in the transdermal delivery of insulin. *Journal of Controlled Release*.

[135] Mathers A. R., Larregina A. T. (2006). Professional antigen-presenting cells of the skin. *Immunologic Research*.

[136] Sugita K., Kabashima K., Atarashi K., Shimauchi T., Kobayashi M., Tokura Y. (2007). Innate immunity mediated by epidermal keratinocytes promotes acquired immunity involving Langerhans cells and T cells in the skin. *Clinical & Experimental Immunology*.

[137] Berger C. L., Vasquez J. G., Shofner J., Mariwalla K., Edelson R. L. (2006). Langerhans cells: mediators of immunity and tolerance. *International Journal of Biochemistry and Cell Biology*.

[138] Romani N., Clausen B. E., Stoitzner P. (2010). Langerhans cells and more: langerin-expressing dendritic cell subsets in the skin. *Immunological Reviews*.

[139] Park J.-H., Allen M. G., Prausnitz M. R. (2005). Biodegradable polymer microneedles: fabrication, mechanics and transdermal drug delivery. *Journal of Controlled Release*.

[140] Lee J. W., Park J.-H., Prausnitz M. R. (2008). Dissolving microneedles for transdermal drug delivery. *Biomaterials*.

[141] Sullivan S. P., Koutsonanos D. G., del Pilar Martin M. (2010). Dissolving polymer microneedle patches for influenza vaccination. *Nature Medicine*.

[142] Matsuo K., Hirobe S., Yokota Y. (2012). Transcutaneous immunization using a dissolving microneedle array protects against tetanus, diphtheria, malaria, and influenza. *Journal of Controlled Release*.

[143] Verbaan F. J., Bal S. M., van den Berg D. J. (2007). Assembled microneedle arrays enhance the transport of compounds varying over a large range of molecular weight across human dermatomed skin. *Journal of Controlled Release*.

[144] Brown T. J., Alcorn D., Fraser J. R. E. (1999). Absorption of hyaluronan applied to the surface of intact skin. *Journal of Investigative Dermatology*.

[145] Brown M. B., Jones S. A. (2005). Hyaluronic acid: a unique topical vehicle for the localized delivery of drugs to the skin. *Journal of the European Academy of Dermatology and Venereology*.

[146] Skehel J. J., Waterfield M. D. (1975). Studies on the primary structure of the influenza virus hemagglutinin. *Proceedings of the National Academy of Sciences of the United States of America*.

[147] Shupp J. W., Nasabzadeh T. J., Rosenthal D. S., Jordan M. H., Fidler P., Jeng J. C. (2010). A review of the local pathophysiologic bases of burn wound progression. *Journal of Burn Care and Research*.

[148] Arturson G. (1996). Pathophysiology of the burn wound and pharmacological treatment. The Rudi Hermans Lecture, 1995. *Burns*.

[149] Schwacha M. G., Thobe B. M., Daniel T., Hubbard W. J. (2010). Impact of thermal injury on wound infiltration and the dermal inflammatory response. *Journal of Surgical Research*.

[150] Pandey M. S., Baggenstoss B. A., Washburn J., Harris E. N., Weigel P. H. (2013). The hyaluronan receptor for endocytosis (HARE) activates NF-*κ*B-mediated gene expression in response to 40–400-kDa, but not smaller or larger, hyaluronans. *Journal of Biological Chemistry*.

[151] Sun L. T., Friedrich E., Heuslein J. L. (2012). Reduction of burn progression with topical delivery of (antitumor necrosis factor-alpha)-hyaluronic acid conjugates. *Wound Repair and Regeneration*.

[152] Le Bourlais C., Acar L., Zia H., Sado P. A., Needham T., Leverge R. (1998). Ophthalmic drug delivery systems—recent advances. *Progress in Retinal and Eye Research*.

[153] Graue E. L., Polack F. M., Balazs E. A. (1980). The protective effect of Na-hyaluronate to corneal endothelium. *Experimental Eye Research*.

[154] Gurny R., Ryser J. E., Tabatabay C., Martenet M., Edman P., Camber O. (1990). Precorneal residence time in humans of sodium hyaluronate as measured by gamma scintigraphy. *Graefe's Archive for Clinical and Experimental Ophthalmology*.

[155] Gomes J. A. P., Amankwah R., Powell-Richards A., Dua H. S. (2004). Sodium hyaluronate (hyaluronic acid) promotes migration of human corneal epithelial cells in vitro. *British Journal of Ophthalmology*.

[156] Tani E., Katakami C., Negi A. (2002). Effects of various eye drops on corneal wound healing after superficial keratectomy in rabbits. *Japanese Journal of Ophthalmology*.

[157] Laurent T. C., Laurent U. B. G., Fraser J. R. E. (1996). The structure and function of hyaluronan: an overview. *Immunology & Cell Biology*.

[158] Saettone M. F., Monti D., Torracca M. T., Chetoni P. (1994). Mucoadhesive ophthalmic vehicles: evaluation of polymeric low-viscosity formulations. *Journal of Ocular Pharmacology*.

[159] Camber O., Edman P., Gurny R. (1987). Influence of sodium hyaluronate on the meiotic effect of pilocarpine in rabbits. *Current Eye Research*.

[160] Bucolo C., Mangiafico P. (1999). Pharmacological profile of a new topical pilocarpine formulation. *Journal of Ocular Pharmacology and Therapeutics*.

[161] Bucolo C., Mangiafico S., Spadaro A. (1996). Methylprednisolone delivery by Hyalobend corneal shields and its effects on rabbit ocular inflammation. *Journal of Ocular Pharmacology and Therapeutics*.

[162] Herrero-Vanrell R., Fernandez-Carballido A., Frutos G., Cadórniga R. (2000). Enhancement of the mydriatic response to tropicamide by bioadhesive polymers. *Journal of Ocular Pharmacology and Therapeutics*.

[163] Gandolfi S. A., Massari A., Orsoni J. G. (1992). Low-molecular-weight sodium hyaluronate in the treatment of bacterial corneal ulcers. *Graefe's Archive for Clinical and Experimental Ophthalmology*.

[164] Cho K. Y., Chung T. W., Kim B. C. (2003). Release of ciprofloxacin from poloxamer-graft-hyaluronic acid hydrogels in vitro. *International Journal of Pharmaceutics*.

[165] Choi K. Y., Saravanakumar G., Park J. H., Park K. (2012). Hyaluronic acid-based nanocarriers for intracellular targeting: interfacial interactions with proteins in cancer. *Colloids and Surfaces B: Biointerfaces*.

[166] Pouyani T., Prestwich G. D. (1994). Functionalized derivatives of hyaluronic acid oligosaccharides: drug carriers and novel biomaterials. *Bioconjugate Chemistry*.

[167] Duncan M. B., Liu M., Fox C., Liu J. (2006). Characterization of the *N*-deacetylase domain from the heparan sulfate *N*-deacetylase/*N*-sulfotransferase 2. *Biochemical and Biophysical Research Communications*.

[168] Mochizuki S., Kano A., Shimada N., Maruyama A. (2009). Uptake of enzymatically-digested hyaluronan by liver endothelial cells *in vivo* and *in vitro*. *Journal of Biomaterials Science, Polymer Edition*.

[169] Harris E. N., Kyosseva S. V., Weigel J. A., Weigel P. H. (2007). Expression, processing, and glycosaminoglycan binding activity of the recombinant human 315-kDa Hyaluronic Acid Receptor for Endocytosis (HARE). *The Journal of Biological Chemistry*.

[170] Ruoslahti E., Bhatia S. N., Sailor M. J. (2010). Targeting of drugs and nanoparticles to tumors. *The Journal of Cell Biology*.

[171] Lesley J., Hascall V. C., Tammi M., Hyman R. (2000). Hyaluronan binding by cell surface CD44. *Journal of Biological Chemistry*.

[172] Luo Y., Prestwich G. D. (1999). Synthesis and selective cytotoxicity of a hyaluronic acid-antitumor bioconjugate. *Bioconjugate Chemistry*.

[173] Coradini D., Pellizzaro C., Miglierini G., Daidone M. G., Perbellini A. (1999). Hyaluronic acid as drug delivery for sodium butyrate: improvement of the anti-proliferative activity on a breast-cancer cell line. *International Journal of Cancer*.

[174] Gaffney J., Matou-Nasri S., Grau-Olivares M., Slevin M. (2010). Therapeutic applications of hyaluronan. *Molecular BioSystems*.

[175] Plattt V. M., Szoka F. C. (2008). Anticancer therapeutics: targeting macromolecules and nanocarriers to hyaluronan or CD44, a hyaluronan receptor. *Molecular Pharmaceutics*.

[176] Luo Y., Ziebell M. R., Prestwich G. D. (2000). A hyaluronic acid—taxol antitumor bioconjugate targeted to cancer cells. *Biomacromolecules*.

[177] Rosato A., Banzato A., de Luca G. (2006). HYTAD1-p20: a new paclitaxel-hyaluronic acid hydrosoluble bioconjugate for treatment of superficial bladder cancer. *Urologic Oncology*.

[178] Serafino A., Zonfrillo M., Andreola F. (2011). CD44-targeting for antitumor drug delivery: a new SN-38-hyaluronan bioconjugate for locoregional treatment of peritoneal carcinomatosis. *Current Cancer Drug Targets*.

[179] Bassi P. F., Volpe A., D'Agostino D. (2011). Paclitaxel-hyaluronic acid for intravesical therapy of bacillus Calmette-Guerin refractory carcinoma in situ of the bladder: results of a phase I study. *Journal of Urology*.

[180] Montagner I. M., Banzato A., Zuccolotto G. (2013). Paclitaxel-hyaluronan hydrosoluble bioconjugate: mechanism of action in human bladder cancer cell lines. *Urologic Oncology*.

[181] Luo Y., Kirker K. R., Prestwich G. D. (2000). Cross-linked hyaluronic acid hydrogel films: new biomaterials for drug delivery. *Journal of Controlled Release*.

[182] Tringali G., Bettella F., Greco M. C., Campisi M., Renier D., Navarra P. (2012). Pharmacokinetic profile of Oncofid-S after intraperitoneal and intravenous administration in the rat. *Journal of Pharmacy and Pharmacology*.

[183] Cohen S. M., Rockefeller N., Mukerji R. (2013). Efficacy and toxicity of peritumoral delivery of nanoconjugated cisplatin in an *in vivo* murine model of head and neck squamous cell carcinoma. *JAMA Otolaryngology: Head and Neck Surgery*.

[184] Brown T. J. (2008). The development of hyaluronan as a drug transporter and excipient for chemotherapeutic drugs. *Current Pharmaceutical Biotechnology*.

[185] Rosenthal M. A., Gibbs P., Brown T. J. (2005). Phase I and pharmacokinetic evaluation of intravenous hyaluronic acid in combination with doxorubicin or 5-fluorouracil. *Chemotherapy*.

[186] Gibbs P., Brown T. J., Ng R. (2009). A pilot human evaluation of a formulation of irinotecan and hyaluronic acid in 5-fluorouracil-refractory metastatic colorectal cancer patients. *Chemotherapy*.

[187] Varghese O. P., Sun W., Hilborn J., Ossipov D. A. (2009). In situ cross-linkable high molecular weight hyaluronan-bisphosphonate conjugate for localized delivery and cell-specific targeting: a hydrogel linked prodrug approach. *Journal of the American Chemical Society*.

[188] Di Meo C., Panza L., Capitani D. (2007). Hyaluronan as carrier of carboranes for tumor targeting in boron neutron capture therapy. *Biomacromolecules*.

[189] di Meo C., Panza L., Campo F. (2008). Novel types of carborane-carrier hyaluronan derivatives via ‘click chemistry’. *Macromolecular Bioscience*.

[190] Jain A., Jain S. K. (2008). In vitro and cell uptake studies for targeting of ligand anchored nanoparticles for colon tumors. *European Journal of Pharmaceutical Sciences*.

[191] Jain A., Jain S. K., Ganesh N., Barve J., Beg A. M. (2010). Design and development of ligand-appended polysaccharidic nanoparticles for the delivery of oxaliplatin in colorectal cancer. *Nanomedicine: Nanotechnology, Biology, and Medicine*.

[192] Bachar G., Cohen K., Hod R. (2011). Hyaluronan-grafted particle clusters loaded with Mitomycin C as selective nanovectors for primary head and neck cancers. *Biomaterials*.

[193] Peer D., Margalit R. (2004). Loading mitomycin C inside long circulating hyaluronan targeted nano-liposomes increases its antitumor activity in three mice tumor models. *International Journal of Cancer*.

[194] Peer D., Margalit R. (2004). Tumor-targeted hyaluronan nanoliposomes increase the antitumor activity of liposomal doxorubicin in syngeneic and human xenograft mouse tumor models. *Neoplasia*.

[195] Surace C., Arpicco S., Dufaÿ-Wojcicki A. (2009). Lipoplexes targeting the CD44 hyaluronic acid receptor for efficient transfection of breast cancer cells. *Molecular Pharmaceutics*.

[196] Eliaz R. E., Szoka F. C. (2001). Liposome-encapsulated doxorubicin targeted to CD44: a strategy to kill CD44-overexpressing tumor cells. *Cancer Research*.

[197] Ruhela D., Riviere K., Szoka F. C. (2006). Efficient synthesis of an aldehyde functionalized hyaluronic acid and its application in the preparation of hyaluronan-lipid conjugates. *Bioconjugate Chemistry*.

[198] Dufaÿ Wojcicki A., Hillaireau H., Nascimento T. L. (2012). Hyaluronic acid-bearing lipoplexes: physico-chemical characterization and in vitro targeting of the CD44 receptor. *Journal of Controlled Release*.

[199] Liu Y., Sun J., Cao W. (2011). Dual targeting folate-conjugated hyaluronic acid polymeric micelles for paclitaxel delivery. *International Journal of Pharmaceutics*.

[200] Qiu L., Li Z., Qiao M. (2014). Self-assembled pH-responsive hyaluronic acid-g-poly(l-histidine) copolymer micelles for targeted intracellular delivery of doxorubicin. *Acta Biomaterialia*.

[201] Zhang Y., Zhang H., Wang X., Wang J., Zhang X., Zhang Q. (2012). The eradication of breast cancer and cancer stem cells using octreotide modified paclitaxel active targeting micelles and salinomycin passive targeting micelles. *Biomaterials*.

[202] Lee H., Mok H., Lee S., Oh Y.-K., Park T. G. (2007). Target-specific intracellular delivery of siRNA using degradable hyaluronic acid nanogels. *Journal of Controlled Release*.

[203] Pellegrino T., Kudera S., Liedl T., Javier A. M., Manna L., Parak W. J. (2005). On the development of colloidal nanoparticles towards multifunctional structures and their possible use for biological applications. *Small*.

[204] Tenne R. (2006). Inorganic nanotubes and fullerene-like nanoparticles. *Nature Nanotechnology*.

[205] Baker S. N., Baker G. A. (2010). Luminescent carbon nanodots: emergent nanolights. *Angewandte Chemie*.

[206] Geim A. K. (2009). Graphene: status and prospects. *Science*.

[207] Lee M.-Y., Yang J.-A., Jung H. S. (2012). Hyaluronic acid-gold nanoparticle/interferon *α* complex for targeted treatment of hepatitis C virus infection. *ACS Nano*.

[208] Kumar A., Sahoo B., Montpetit A., Behera S., Lockey R. F., Mohapatra S. S. (2007). Development of hyaluronic acid-Fe_2_O_3_ hybrid magnetic nanoparticles for targeted delivery of peptides. *Nanomedicine: Nanotechnology, Biology, and Medicine*.

[209] Lu J., Liong M., Li Z., Zink J. I., Tamanoi F. (2010). Biocompatibility, biodistribution, and drug-delivery efficiency of mesoporous silica nanoparticles for cancer therapy in animals. *Small*.

[210] Cho H.-J., Yoon H. Y., Koo H. (2011). Self-assembled nanoparticles based on hyaluronic acid-ceramide (HA-CE) and Pluronic for tumor-targeted delivery of docetaxel. *Biomaterials*.

[211] Lesley J., English N., Charles C., Hyman R. (2000). The role of the CD44 cytoplasmic and transmembrane domains in constitutive and inducible hyaluronan binding. *European Journal of Immunology*.

[212] Lesley J., Hyman R. (1992). CD44 can be activated to function as an hyaluronic acid receptor in normal murine T cells. *European Journal of Immunology*.

[213] Culty M., Nguyen H. A., Underhill C. B. (1992). The hyaluronan receptor (CD44) participates in the uptake and degradation of hyaluronan. *The Journal of Cell Biology*.

[214] Cichy J., Puré E. (2003). The liberation of CD44. *Journal of Cell Biology*.

[215] Allouche M., Charrad R. S., Bettaieb A., Greenland C., Grignon C., Smadja- Joffe F. (2000). Ligation of the CD44 adhesion molecule inhibits drug-induced apoptosis in human myeloid leukemia cells. *Blood*.

[216] Legras S., Lévesque J.-P., Charrad R. (1997). CD44-mediated adhesiveness of human hematopoietic progenitors to hyaluronan is modulated by cytokines. *Blood*.

[217] Katoh S., Zheng Z., Oritani K., Shimozato T., Kincade P. W. (1995). Glycosylation of CD44 negatively regulates its recognition of hyaluronan. *Journal of Experimental Medicine*.

[218] He Q., Lesley J., Hyman R., Ishihara K., Kincade P. W. (1992). Molecular isoforms of murine CD44 and evidence that the membrane proximal domain is not critical for hyaluronate recognition. *Journal of Cell Biology*.

[219] Sleeman J., Rudy W., Hofmann M., Moll J., Herrlich P., Ponta H. (1996). Regulated clustering of variant CD44 proteins increases their hyaluronate binding capacity. *Journal of Cell Biology*.

[220] Sleeman J. P., Arming S., Moll J. F. (1996). Hyaluronate-independent metastatic behavior of CD44 variant-expressing pancreatic carcinoma cells. *Cancer Research*.

[221] Sleeman J. P., Kondo K., Moll J., Ponta H., Herrlich P. (1997). Variant exons v6 and v7 together expand the repertoire of glycosaminoglycans bound by CD44. *The Journal of Biological Chemistry*.

[222] Vercruysse K. P., Prestwich G. D., Kuo J.-W. (1998). Hyaluronate derivatives in drug delivery. *Critical Reviews in Therapeutic Drug Carrier Systems*.

[223] Jaracz S., Chen J., Kuznetsova L. V., Ojima I. (2005). Recent advances in tumor-targeting anticancer drug conjugates. *Bioorganic and Medicinal Chemistry*.

[224] Pouyani T., Prestwich G. D. (1994). Biotinylated hyaluronic acid: a new tool for probing hyaluronate-receptor interactions. *Bioconjugate Chemistry*.

[225] Prestwich G. D., Marecak D. M., Marecek J. F., Vercruysse K. P., Ziebell M. R. (1998). Controlled chemical modification of hyaluronic acid: synthesis, applications, and biodegradation of hydrazide derivatives. *Journal of Controlled Release*.

[226] Liao Y.-H., Jones S. A., Forbes B., Martin G. P., Brown M. B. (2005). Hyaluronan: pharmaceutical characterization and drug delivery. *Drug Delivery*.

[227] Yadav A. K., Mishra P., Agrawal G. P. (2008). An insight on hyaluronic acid in drug targeting and drug delivery. *Journal of Drug Targeting*.

[228] Afratis N., Gialeli C., Nikitovic D. (2012). Glycosaminoglycans: key players in cancer cell biology and treatment. *FEBS Journal*.

[229] Orian-Rousseau V. (2010). CD44, a therapeutic target for metastasising tumours. *European Journal of Cancer*.

[230] Zöller M. (2011). CD44: can a cancer-initiating cell profit from an abundantly expressed molecule?. *Nature Reviews Cancer*.

[231] Heider K.-H., Kuthan H., Stehle G., Munzert G. (2004). CD44v6: a target for antibody-based cancer therapy. *Cancer Immunology, Immunotherapy*.

[232] Zeilstra J., Joosten S. P. J., van Andel H. (2014). Stem cell CD44v isoforms promote intestinal cancer formation in Apc(min) mice downstream of Wnt signaling. *Oncogene*.

[233] Stauder R., Eisterer W., Thaler J., Gunthert U. (1995). CD44 variant isoforms in non-Hodgkin's lymphoma: a new independent prognostic factor. *Blood*.

[234] Todaro M., Alea M. P., di Stefano A. B. (2007). Colon cancer stem cells dictate tumor growth and resist cell death by production of interleukin-4. *Cell Stem Cell*.

[235] Guo W., Frenette P. S. (2014). Alternative CD44 splicing in intestinal stem cells and tumorigenesis. *Oncogene*.

[236] Kainz C., Kohlberger P., Sliutz G. (1995). Splice variants of CD44 in human cervical cancer stage IB to IIB. *Gynecologic Oncology*.

[237] Kainz C., Kohlberger P., Tempfer C. (1995). Prognostic value of CD44 splice variants in human stage III cervical cancer. *European Journal of Cancer Part A: General Topics*.

[238] Hsieh H.-F., Yu J.-C., Ho L.-I., Chiu S.-C., Harn H.-J. (1999). Molecular studies into the role of CD44 variants in metastasis in gastric cancer. *Journal of Clinical Pathology: Molecular Pathology*.

[239] Shtivelman E., Bishop J. M. (1991). Expression of CD44 is repressed in neuroblastoma cells. *Molecular and Cellular Biology*.

[240] de Marzo A. M., Bradshaw C., Sauvageot J., Epstein J. I., Miller G. J. (1998). CD44 and CD44v6 downregulation in clinical prostatic carcinoma: relation to Gleason grade and cytoarchitecture. *The Prostate*.

[241] Seiter S., Arch R., Reber S. (1993). Prevention of tumor metastasis formation by anti-variant CD44. *Journal of Experimental Medicine*.

[242] Colnot D. R., Wilhelm A. J., Cloos J. (2001). Evaluation of limited blood sampling in a preceding 99mTc-labeled diagnostic study to predict the pharmacokinetics and myelotoxicity of 186Re-cMAb U36 radioimmunotherapy. *Journal of Nuclear Medicine*.

[243] De Bree R., Roos J. C., Quak J. J., Den Hollander W., Snow G. B., Van Dongen G. A. M. S. (1995). Radioimmunoscintigraphy and biodistribution of technetium-99m-labeled monoclonal antibody U36 in patients with head and neck cancer. *Clinical Cancer Research*.

[244] de Bree R., Roos J. C., Quak J. J. (1995). Biodistribution of radiolabeled monoclonal antibody E48 IgG and F(ab′)2 in patients with head and neck cancer. *Clinical Cancer Research*.

[245] Börjesson P. K. E., Postema E. J., Roos J. C. (2003). Phase I therapy study with 186Re-labeled humanized monoclonal antibody BIWA 4 (Bivatuzumab) in patients with head and neck squamous cell carcinoma. *Clinical Cancer Research*.

[246] Colnot D. R., Roos J. C., de Bree R. (2003). Safety, biodistribution, pharmacokinetics, and immunogenicity of ^99m^Tc-labeled humanized monoclonal antibody BIWA 4 (bivatuzumab) in patients with squamous cell carcinoma of the head and neck. *Cancer Immunology, Immunotherapy*.

[247] Tijink B. M., Buter J., de Bree R. (2006). A phase I dose escalation study with anti-CD44v6 bivatuzumab mertansine in patients with incurable squamous cell carcinoma of the head and neck or esophagus. *Clinical Cancer Research*.

[248] Urakawa H., Nishida Y., Knudson W. (2012). Therapeutic potential of hyaluronan oligosaccharides for bone metastasis of breast cancer. *Journal of Orthopaedic Research*.

[249] Misra S., Ghatak S., Zoltan-Jones A., Toole B. P. (2003). Regulation of multidrug resistance in cancer cells by hyaluronan. *The Journal of Biological Chemistry*.

[250] St. Croix B., Rak J. W., Kapitain S., Sheehan C., Graham C. H., Kerbel R. S. (1996). Reversal by hyaluronidase of adhesion-dependent multicellular drug resistance in mammary carcinoma cells. *Journal of the National Cancer Institute*.

[251] St. Croix B., Man S., Kerbel R. S. (1998). Reversal of intrinsic and acquired forms of drug resistance by hyaluronidase treatment of solid tumors. *Cancer Letters*.

[252] Lokeshwar V. B., Estrella V., Lopez L. (2006). HYAL1-v1, an alternatively spliced variant of HYAL1 hyaluronidase: a negative regulator of bladder cancer. *Cancer Research*.

[253] Kursa M., Walker G. F., Roessler V. (2003). Novel shielded transferrin-polyethylene glycol-polyethylenimine/DNA complexes for systemic tumor-targeted gene transfer. *Bioconjugate Chemistry*.

[254] Bellocq N. C., Pun S. H., Jensen G. S., Davis M. E. (2003). Transferrin-containing, cyclodextrin polymer-based particles for tumor-targeted gene delivery. *Bioconjugate Chemistry*.

[255] Ghatak S., Hascall V. C., Berger F. G. (2008). Tissue-Specific shRNA delivery: a novel approach for gene therapy in cancer. *Connective Tissue Research*.

[256] Song G., Liao X., Zhou L., Wu L., Feng Y., Han Z. C. (2004). HI44a, an anti-CD44 monoclonal antibody, induces differentiation and apoptosis of human acute myeloid leukemia cells. *Leukemia Research*.

[257] Riechelmann H., Sauter A., Golze W. (2008). Phase I trial with the CD44v6-targeting immunoconjugate bivatuzumab mertansine in head and neck squamous cell carcinoma. *Oral Oncology*.

[258] Koppe M., van Schaijk F., Roos J. (2004). Safety, pharmacokinetics, immunogenicity, and biodistribution of 186Re-labeled humanized monoclonal antibody BIWA 4 (Bivatuzumab) in patients with early-stage breast cancer. *Cancer Biotherapy and Radiopharmaceuticals*.

[259] Toole B. P., Ghatak S., Misra S. (2008). Hyaluronan oligosaccharides as a potential anticancer therapeutic. *Current Pharmaceutical Biotechnology*.

[260] Paul C. P., Good P. D., Winer I., Engelke D. R. (2002). Effective expression of small interfering RNA in human cells. *Nature Biotechnology*.

[261] Raper S. E., Chirmule N., Lee F. S. (2003). Fatal systemic inflammatory response syndrome in a ornithine transcarbamylase deficient patient following adenoviral gene transfer. *Molecular Genetics and Metabolism*.

[262] Ailles L. E., Weissman I. L. (2007). Cancer stem cells in solid tumors. *Current Opinion in Biotechnology*.

[263] Lobo N. A., Shimono Y., Qian D., Clarke M. F. (2007). The biology of cancer stem cells. *Annual Review of Cell and Developmental Biology*.

[264] Dalerba P., Cho R. W., Clarke M. F. (2007). Cancer stem cells: models and concepts. *Annual Review of Medicine*.

